# Cross‐Site Generalization of CNN‐Based $B_{1}^{+}$ Mapping in UHF MRI

**DOI:** 10.1002/nbm.70263

**Published:** 2026-04-06

**Authors:** Kimon Hadjikiriakos, Felix Krüger, Felix Frederik Zimmermann, Johannes A. Grimm, Constantin Schorling, Max Lutz, Simon Schmidt, Layla Tabea Riemann, Katja Degenhardt, Tobias Schäffter, Mark E. Ladd, Gregory J. Metzger, Christoph Stefan Aigner, Sebastian Schmitter

**Affiliations:** ^1^ Physikalisch‐Technische Bundesanstalt Berlin Germany; ^2^ Medical Physics in Radiology German Cancer Research Center (DKFZ) Heidelberg Germany; ^3^ Faculty of Physics and Astronomy Heidelberg University Heidelberg Germany; ^4^ Center for Magnetic Resonance Research University of Minnesota Minneapolis Minnesota USA; ^5^ Institute for Applied Medical Informatics University Medical Center Hamburg‐Eppendorf (UKE) Hamburg Germany; ^6^ Max Planck Research Group MR Physics, Max Planck Institute for Human Development Berlin Germany

**Keywords:** 7T, $B_{1}^{+}$ mapping, brain, deep learning, model generalization, model transferability, parallel transmission

## Abstract

Convolutional neural networks (CNNs) can rapidly predict channel‐wise $B_{1}^{+}$ maps from 7T localizer images, reducing acquisition time to seconds. This paper investigates if a CNN trained on one site's data can generalize to predict $B_{1}^{+}$ maps for brain imaging at unseen sites supporting the feasibility of a universal network for subject‐specific $B_{1}^{+}$‐ mapping. We evaluated a U‐Net CNN cross‐site generalization by training on datasets from two different 7T sites and testing its performance across three 7T sites (1 additional testing site) to assess robustness, adaptability, and generalization. The study design included both commercially same systems and the identical physical hardware unit transported between two sites, enabling a more insightful attribution of performance differences to either hardware issues or dataset‐specific variations.

To assess prediction quality, we examined magnitude/phase images, error maps, correlation plots, Pearson coefficients, and residual spread plots. Quantitative evaluation included RMSE and SSIM scores. Finally, we calculated 4 kT‐points pulses with both on‐site and cross‐site CNNs to evaluate the effectiveness of the obtained $B_{1}^{+}$ maps for parallel‐transmission (pTx).

While on‐site $B_{1}^{+}$ transversal magnitude RMSE scores were as low as 3.0% and 3.1% for the two CNNs, their respective transfer yielded 3.6% and 4.1%. The dynamic pTx‐application showed a CV of 6.5% when using $B_{1}^{+}$ maps predicted by a network trained on its own site. The transfer case, using a map predicted from a network trained on a different site, yielded an increased CV of 13.7%.

Although cross‐site applications introduced larger deviations, the predicted maps remained qualitatively plausible and enabled practical use cases, such as calculating dynamic‐pTx. These findings support the potential of cross‐site training, suggesting CNNs trained at one site may generalize sufficiently to unseen sites without additional adjustments. This strengthens the feasibility of a transferable training approach where a single network could be deployed across different institutions without extensive retraining.

## Graphical abstract

The study demonstrates that CNN%‐based $B_{1}^{+}$ mapping transfers across sites with only moderate accuracy loss: while on‐site RMSEs were as low as ~3%, cross‐site predictions increased errors but remained qualitatively robust. Despite these deviations, cross‐site maps successfully enabled dynamic 4kT‐points pulses, achieving flip‐angle uniformity with CVs around 13%‐15%, compared to 6%‐7% on‐site. These findings highlight that transferable CNN models can provide practical $B_{1}^{+}$ maps for pTx, supporting scalable calibration‐free workflows across different UHF MRI sites.

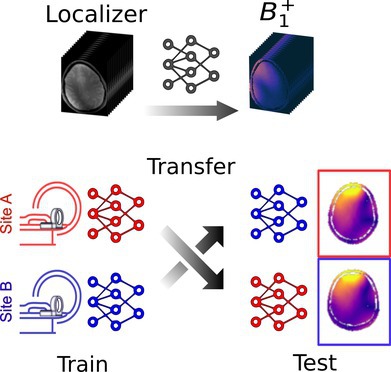

**Abbreviations**
AFI: actual flip‐angle imaging
$B_{1}^{+}$
: transmit magnetic field
CNR: contrast‐to‐noise ratio
CNN/CNNs: convolutional neural network(s)
CP^+^
: circularly polarized mode
CV: coefficient of variation
DAM: double angle method
DL: deep learning
DREAM: dual refocusing echo acquisition mode
FA(s): flip angle(s)
GANs: generative adversarial networks
GRE: gradient echo
GT: ground truth
GTA/GTB: ground truth data from Site A/Site B
KDE: kernel density estimation
NN: neural network
PR: network predictions
PSNR: peak signal‐to‐noise ratio
RF: radiofrequency
RMSE: root‐mean‐square error
RSS: root‐sum‐of‐squares
SAR: specific absorption rate
SNR: signal‐to‐noise ratio
SSIM: structural similarity index measure
STA: small tip angle
TA: acquisition time
TE: echo time
TR: repetition time
Tx/Rx: transmit/receive
UHF: ultrahigh field
UP: universal pulses

## 1 Introduction

Ultrahigh field (UHF) MRI offers inherent advantages, such as higher signal‐to‐noise ratio (SNR) and, for certain applications alike $T_{2}^{*}$ weighted imaging, improved contrast‐to‐noise ratio (CNR) [1]. The increased SNR/CNR enable improved spatial, temporal, and spectral resolutions, which are beneficial for a wide range of applications [2], including neurodegenerative diseases and epilepsy [3, 4]. However, UHF MRI is often challenged by spatial variations in the transmit magnetic field ($B_{1}^{+}$), leading to spatially dependent flip angles (FAs) and, consequently, undesirable contrast variations. Achieving homogeneous $B_{1}^{+}$ fields is essential to unlock the potential of UHF MRI and deliver high‐quality diagnostic information.

Parallel transmission (pTx) [5, 6, 7, 8, 9, 10, 11] addresses spatial FA variability by utilizing multichannel radiofrequency (RF) transmit coils with either common (RF shimming or static pTx) or channel specific RF pulses (dynamic pTx) [12, 13]. These pTx pulses are typically generated either through subject‐specific tailored optimization or by precomputed universal pulses (UPs) applicable to certain target regions in a range of subjects [14, 15]. Although UPs require no subject‐specific calibration, subject‐tailored pTx approaches generally achieve superior performance [14, 15, 16]. This calibration step includes the measurement of channel‐wise $B_{1}^{+}$ maps for each subject, which is often time‐consuming, ranging from tens of seconds to multiple minutes [12, 17, 18, 19], depending on the technique, coverage, resolution, number of Tx channels, and other parameters.

Several established methods for $B_{1}^{+}$ mapping [20, 21, 22] employ diverse encoding techniques, such as double angle method (DAM) [20], AFI [21, 23], phase shifts originating from off‐resonant RF pulses [22], and stimulated echoes [24], have been validated across various field strengths and anatomical regions. Fast mapping methods have emerged, for instance, SA2RAGE [25] that facilitates whole‐brain 3D mapping in under 20 seconds, or DREAM [24, 26], which achieves subsecond per‐slice acquisition. In addition, Kent et al. [27] presented a fast 3D $B_{1}^{+}$ mapping enabling accurate whole‐heart $B_{1}^{+}$ mapping at 7T within a single breath‐hold by eliminating idle delays and reducing $T_{1}$ bias.

To further accelerate $B_{1}^{+}$ mapping, deep learning (DL)–based techniques [28, 29] have emerged as a promising avenue. Zimmermann et al. [30] introduced a semisupervised learning strategy for joint $T_{1},B_{0}$, and $B_{1}^{+}$ mapping, incorporating spatial regularization into quantitative reconstruction (at 3T). Eberhardt et al. [31] demonstrated that generative adversarial networks (GANs) can synthesize $B_{1}^{+}$ maps from anatomical scans, accelerating the design of pTx pulses at UHF. Wu et al. [32] proposed a deep CNN that predicts subject‐specific SAR distributions from structural MRIs and $B_{1}^{+}$ maps for 7T applications. Plumley et al. [33] developed a NN to estimate $B_{1}^{+}$ fields in real‐time under head motion, enabling dynamic recalibration of pTx pulses. More recently, Krüger et al. [34] demonstrated fast DL‐based $B_{1}^{+}$ mapping enabling rapid pTx calibration for 7T cardiac applications. Their follow‐up work in the brain [35] demonstrated whole‐brain $B_{1}^{+}$ predictions from multislice localizers using complex‐valued CNNs, achieving robust performance across multiple slice orientations. These results highlight the feasibility of CNNs for rapid pTx calibration, particularly where $B_{1}^{+}$ mapping is a time‐limiting step and enables the derivation of $B_{1}^{+}$ maps in subseconds timescales from routinely acquired localizer data [34, 35].

Despite these substantial advances, the CNN‐based $B_{1}^{+}$ mapping techniques also have limitations. For instance, such networks have been trained for a given target region and RF coil, and they necessitate an extensive amount of training data, often acquired using custom hardware configurations. This makes it time‐consuming to build a sufficiently large training library. The library must also reflect the substantial anatomical variability, especially seen in human body imaging [34, 36], as well as coil position differences. Therefore, a collaborative data collection across multiple UHF sites would distribute the effort of building a robust training library. Additionally, if trained CNNs could be transferred between sites, it would eliminate the need for a site‐specific network. While an identical coil would be likely required, as the networks are highly hardware‐dependent, this is often the case in head applications, where a widely used commercial 8Tx/32Rx coil is available. However, the intersite transferability of trained models remains unclear.

This study aims to address the challenges of model generalization by quantifying the impact of intersite variability on CNN‐based $B_{1}^{+}$ prediction accuracy. Furthermore, we investigate whether such models can be used interchangeably across sites without requiring additional training or domain adaptation. To minimize confounding factors, the study focuses on 7T head MRI using a broadly used commercial pTx coil. Data were acquired using either the identical commercial coil used at different UHF sites or a duplicate at different UHF sites in combination with scanners from the same vendor. The robustness and generalizability of the CNNs were assessed via in vivo cross‐site study design, where networks trained at one site predicted $B_{1}^{+}$ magnitudes and phases from data collected at another site.

## 2 Methods

### 2.1 Subjects and MR Scanner Hardware

**FIGURE 1:**
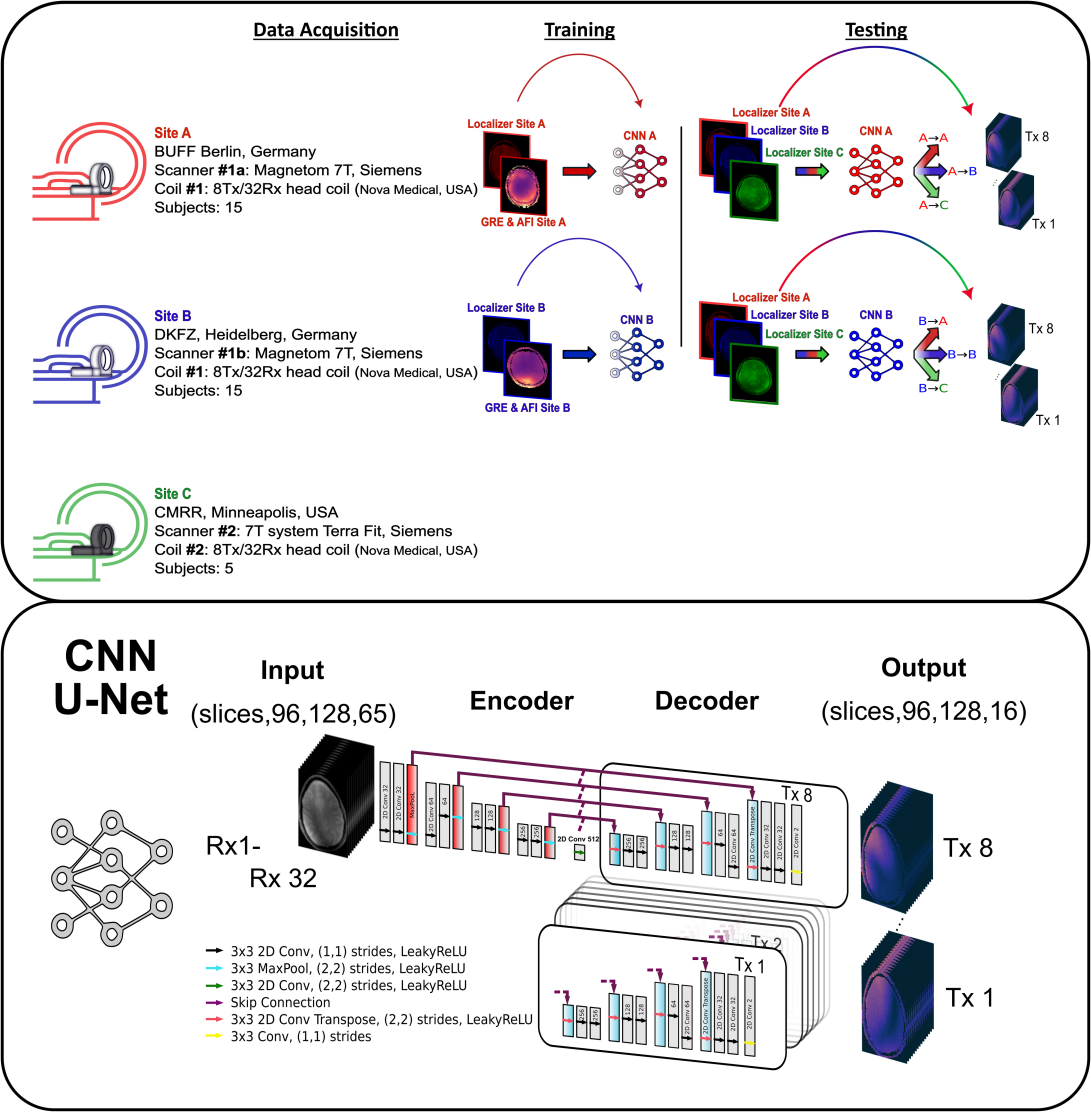
Schematic overview of the study design. Data were collected from three testing sites: Site A (red) and Site B (blue), each contributing 15 subjects acquired on a (different) Siemens Magnetom 7T scanner using coil #1; and Site C (green), with five subjects scanned on a Siemens 7T Terra Fit using coil #2. Data from Sites A and B were used to train separate CNN U‐Net models to predict $B_{1}^{+}$ maps from GRE input, resulting in two trained networks (red: Site A CNN; blue: Site B CNN). Both networks were then evaluated on data from all three sites (A, B, and C). The lower panel illustrates the CNN architecture, featuring a single encoder and eight decoder heads, corresponding to the eight complex valued Tx channels.

MRI data were collected as depicted in Figure 1 at three UHF facilities using two different 7T systems (2 different classic 7T Magnetom; 1 7T Terra Fit; Siemens Healthineers, Germany) and two versions of the same 8Tx/32Rx head coil (Nova Medical, Wilmington, USA). Site A (Berlin, Germany) collected data from 15 subjects (sex: F: 6; M: 9| average age: 38.4 years) using a classic 7T Magnetom and coil #1. Site B (Heidelberg, Germany) also collected data from 15 subjects (sex: F: 4; M: 11|average age: 25 years), using a different classic 7T Magnetom but used the identical coil #1 (hardware unit transported between two sites). Site C (Minneapolis, USA) provided data from five subjects (sex: F: 2; M: 3|average age: 46.4 years), using a 7T Terra Fit with coil #2. Informed consent was obtained from all volunteers, and the study received approval from the local institutional ethics boards.

### 2.2 MRI Data Acquisition and Data Processing

For each subject, 2D multislice GRE data were acquired in all three anatomical orientations (transversal, sagittal, and coronal) using the following parameters: TE/TR = 4/8.6 ms; resolution = 2 × 2 × 4 mm^3^, slices = 15 (transversal/sagittal) and 21 (coronal), slice gap = 100%, matrix = 128 × 96, FOV = 256 × 192 mm^2^, TA = 15 s, nominal FA = 10°. Two sets of GRE images were acquired with an 80 V fixed reference voltage per Tx channel: one with all channels active in CP^+^ mode (used as localizer), and a second set with each channel activated individually (used for $B_{1}^{+}$ mapping). In addition, matching AFI datasets were acquired in CP^+^ mode with TR_1_/TR_2_ = 25/125 ms, TE = 1.9 ms, resolution = 2x2x4 mm^3^, matrix = 128 × 96 × 64, FOV = 256 × 192 × 256 mm^3^, TA = 9:30 min, nominal FA = 60°.

The GRE scan obtained in CP^+^ mode served as localizer, using all 32 Rx channel‐wise complex data and the root sum of squares magnitude image as input to the CNNs (Figure 1). Spatially resolved relative, Tx‐channel‐wise (normalized to 90°) $B_{1}^{+}$ maps were acquired with a hybrid approach [37, 38] as unbiased $B_{1}^{+}$ reference [37], serving as training targets. Details of the preprocessing steps are described in previous works [34, 35] and are summarized in Supplementary Material (I.1. and I.2).

Two CNNs were trained independently (c.f., Figure 1): CNN A using 12 subjects acquired at Site A with coil #1, and CNN B using 12 subjects from Site B, acquired with the identical coil (#1). Both models were then tested on the remaining unseen data (3 subjects) from their own Site and the opposite Site A/Site B as well as Site C (where coil #2 was employed) to assess the generalization performance and robustness under domain shift conditions. The bidirectionality and transferability of evaluating both A → B (CNN trained on data from Site A, tested on data from Site B) and B → A (CNN trained on data from Site B, tested on data from Site A) performance was qualitatively and quantitatively assessed by different metrics including RMSE, SSIM, and PSNR, as well as through a dynamic pTx application using the predicted $B_{1}^{+}$ maps.

A compact notation was used to indicate the CNN and the input data used to predict $B_{1}^{+}$ maps. This notation is structured as follows: CNN trained with data from Site X applied to input data (localizer) from Site Y will be denoted as X → Y. Thus, for example, A → B denotes a CNN trained on site A (CNN A) applied on unseen input data from site B to predict $B_{1}^{+}$ maps from Site B. We will consider the following cases: A → A, A → B, B → B, B → A, A → C, and B → C. To enhance clarity, a consistent color‐coding scheme is used in the figures: red for Site A (for both input data and trained CNN), blue for Site B, and green for Site C.

All CNNs employed a 2D U‐Net architecture with four encoder resolution levels (32, 64, 128, and 256 feature channels) and a 512‐kernel bottleneck. At each resolution level, double convolutional blocks were used consistently in both the encoder and decoder paths.

The selected U‐Net architecture was based on prior works [34, 35], where this configuration demonstrated robust performance for closely related $B_{1}^{+}$ prediction tasks. Using the same architecture allows for a consistent baseline for the present study. The multihead decoder structure, with separate output branches for each Tx channel, was designed to reflect the underlying multichannel 8Tx/32Rx RF hardware configuration, enabling individual channel analysis and direct comparison with measured $B_{1}^{+}$maps.

Each 2D slice was treated as one training sample. The input comprised 32 complex‐valued GRE localizer images from the 32 Rx channels, real and imaginary components plus the root sum of squares (RSS) combined magnitude image were placed in separate channels, yielding 65 real‐valued input channels per slice with input tensor shape (slices, x, y, 65). The target consisted of the corresponding eight Tx $B_{1}^{+}$ maps, which were likewise split into real and imaginary parts, resulting in 16 real‐valued output channels with tensor shape (slices, x, y, 16). The CNN was trained to learn a mapping from the uncombined, normalized GRE signal (input) to the spatially resolved, complex‐valued $B_{1}^{+}$ field estimates (output) across all eight Tx channels. The CNN architecture used throughout was based on a 2D U‐Net with four resolution levels, an initial number of 32 convolutional filters. Each of the eight Tx channels was decoded by a dedicated output branch, resulting in a multihead structure with a shared encoder and replicated decoder paths.

During training, we explored the use of the ⊥‐loss [35, 39, 40] to assess whether it could reduce magnitude bias and improve the fidelity of complex‐valued predictions, in line with our previously used models; however, we did not observe more stable or improved overall performance compared to the loss functions ultimately employed (see Supplementary Tables S1 and S2). Finally, the CNN was trained using the standard L2 loss function (mean squared error), which was also used to evaluate the model on the validation dataset. Their performance was evaluated using two different “Subsets.” Fixed “subsets” involved validating both CNNs on three randomly selected subjects from each training site (A and B), fixed for all evaluations, and five subjects from Site C. For the “Full Set” evaluation, each model was tested on all 15 subjects from the site it was not trained on, three unseen subjects from the site it was trained for, as well as five subjects from Site C.

This U‐Net design led to a total of approximately 29.1 million trainable parameters. Training was performed using the ADAM optimizer with a learning rate of *η* = 1 × 10^−4^, running for 4000 epochs with a batch size of 1. The total number of training samples was 156, on a 24 GB NVIDIA Titan RTX GPU, one epoch took approximately 15 s to complete. Training hyperparameters and number of epochs were determined through parameter sweeps that showed stable convergence during both training and validation.

The network implementation, together with training scripts and a script demonstrating how to use the trained models for inference, as well as the corresponding data, is publicly available via a GitHub and Zenodo repository at the following links: https://github.com/hkimon/B1P_Mapping_CCN.git and https://doi.org/10.5281/zenodo.18338273.

### 2.3 Evaluation Metrics, Statistical Analysis, and Visualization

The network's performance was assessed using the following three quantitative metrics: root mean square error (RMSE), structural similarity index measure (SSIM), and peak signal to noise ratio (PSNR), c.f., Supplementary Material (I.3–I.5). These were used to evaluate the accuracy of the predicted $B_{1}^{+}$ maps by comparing them to ground truth (GT) data. To systematically assess prediction accuracy, GT and PR (network predictions) were compared using absolute and relative differences. Note, here, the term “ground truth” refers to the measured relative B1+ maps, which are inherently subject to noise, processing approximations, and other experimental uncertainties. Details of the different metrics are provided in Supplementary Material (I.3–I.5).

Moreover, several visualization and analysis techniques for evaluating predictive model performance were used, including error maps to highlight significant deviations from ground truth, residual spread plots (modified Bland–Altman) to visualize residual distributions, and correlation plots to assess the relationship between PR and GT. Additionally, distribution plots are used to analyze the spread and consistency of prediction accuracy metrics like SSIM and RMSE across different sites. Additional methodological details, extended statistical analyses, and complete numerical results are provided in the Supplementary Material accompanying this article.

Subject‐specific dynamic pTx pulses [41] based on the PR and GT $B_{1}^{+}$ maps were designed and their pTx application was simulated using the small tip angle (STA) approximation, neglecting relaxation and assuming piece‐wise constant RF and gradient fields, following a spatial‐domain method [5, 42], to assess the impact of the PR quality on resulting excitations. The RF pulses were designed, and applied to the following three different pTx examples: (i) pTx pulses designed using GT from Site A (GTA) and B (GTB), respectively, and applied on the GT, (ii) using PR of CNN A applied to A → A|B → A, and (iii) using PR of CNN B applied to B → B|A → B.

## 3 Results

An extensive ablation study was conducted to assess the impact of loss function (L2, L2 + ⊥), convolution type (real, complex), input dimensionality (2D, 2.5D), and training strategy (full results for complex valued CNNs are provided in the Supplementary Table S2). After systematic evaluation, real‐valued networks trained with an L2 loss were selected for all subsequent training, as they achieved performance that was comparable to more complex configurations while exhibiting consistently stable behavior across sites (Table 1).

**TABLE 1:** Ablation study on architecture, loss function, input dimensionality, and training strategy. All experiments were performed on transversal slices. Values are reported as RMSE ± SD|SSIM ± SD (%). Within each ablation group, boldface values indicate the best‐performing configuration when comparing RMSE and SSIM scores.

ID	Train	Conv. type	Input	Loss	Test A RMSE|SSIM (%)	Test B RMSE|SSIM (%)	Test C RMSE|SSIM (%)
(a) **Baseline**: **single‐site, 2D, real‐valued, L2**							
Ref.	A→	Real	1 slice	$L_{2}$	**3.0** ± 1.6|**90.9** ± 10.5	4.1 ± 1.9|87.7 ± 7.8	3.2 ± 1.2|**92.9** ± 2.6
Ref.	B→	Real	1 slice	$L_{2}$	3.6 ± 1.6|84.7 ± 12.7	**3.1** ± 1.4|**91.3** ± 8.3	**3.1** ± 0.9|89.5 ± 3.6
(b) **Ablation**: loss function (**L2 → L2 + ⊥**)							
AX I	A→	Real	1 slice	$L_{2}\;+\;⊥$	**3.46** ± 1.4|**88.2** ± 11.1	4.35 ± 1.8|85.7 ± 7.9	3.3 ± 0.7|**90.4** ± 2.6
AX I	B→	Real	1 slice	$L_{2}\;+\;⊥$	3.59 ± 1.6|**91.7** ± 8.4	**3.27** ± 1.9|85.0 ± 12.8	**2.94** ± 0.7|90.3 ± 3.1
(c.1) **Ablation**: convolution type **(real → complex, L2)**							
AX II	A→	Complex	1 slice	$L_{2}$	**2.8** ± 1.7**|87.7** ± 6.7	4.4 ± 2.1|82.1 ± 7.2	6.2 ± 3.4|**64.9** ± 6.5
AX II	B→	Complex	1 slice	$L_{2}$	3.65 ± 1.8|82.3 ± 1.8	**3.3** ± 2.1|**90.3** ± 10.8	**6.0** ± 1.6|64.0 ± 6.3
(c.2) **Ablation**: convolution type (**real → complex, L2 + ⊥**)							
AX II	A→	Complex	1 slice	$L_{2}\;+\;⊥$	**2.76** ± 1.6**|89.9** ± 7.2	4.14 ± 2.1|85.0 ± 6.0	**6.0** ± 1.8|**65.4** ± 7.7
AX II	B→	Complex	1 slice	$L_{2}\;+\;⊥$	11.0 ± 6.2|71.6 ± 2.8	**3.30** ± 2.1|**90.8** ± 5.2	9.9 ± 3.5|59.7 ± 9.5
(d) **Ablation**: input dimensionality (**2D → 2.5D**)							
DX	A→	Real	3 slices	$L_{2}$	**2.73** ± 1.3**|91.9** ± 9.4	**3.65** ± 1.0|90.0 ± 2.6	**2.86** ± 0.8|**92.8** ± 2.2
DX	B→	Real	3 slices	$L_{2}$	3.07 ± 1.1|86.1 ± 10.9	3.48 ± 1.4|**92.9** ± 4.5	3.08 ± 0.7|89.6 ± 3.0
(e) **Ablation**: training strategy (**single site → pooled**)							
Pooled	A |B→	Real	1 slice	$L_{2}$	2.78 ± 1.5|90.7 ± 10.9	3.27 ± 1.5|91.7 ± 8.2	2.91 ± 0.9|93.2 ± 2.2

### 3.1 $B_{1}^{+}$ Magnitudes and Phases

**FIGURE 2:**
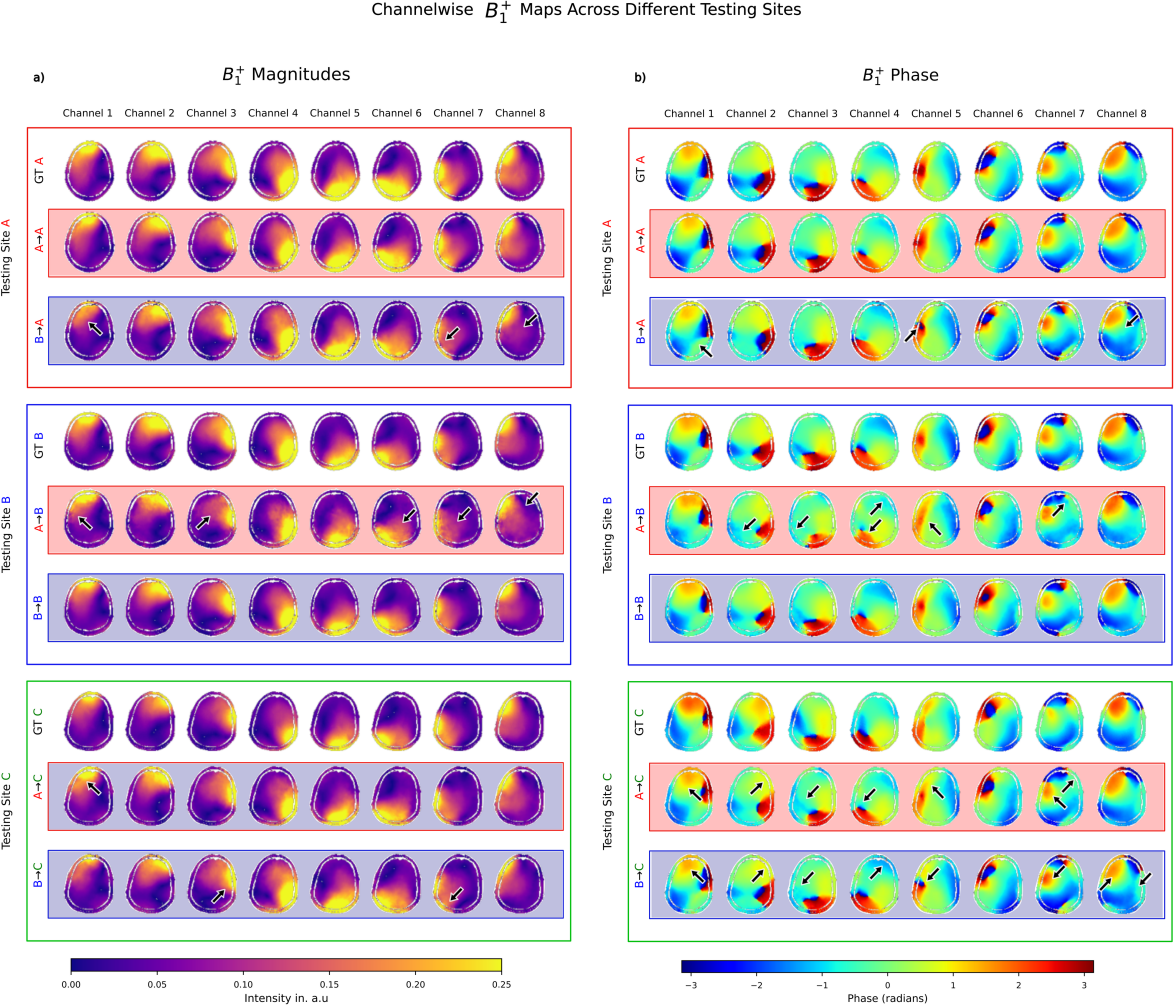
(a) Magnitude of the channel‐wise $B_{1}^{+}$ maps comparing ground truth (GT) and predictions (PR) from sites A, B, and C. Outer bounding box colors indicate testing sites; inner box colors indicate CNNs. All examples show the central transverse slice out of the 3D volume. CNNs perform best on their own site, with smoother magnitude maps. A → C outperforms B → C on Site C. Notable prediction artifacts appear in rows 3 and 5, 8, and 9 and columns 1–3 and 6–7. The arrows indicate areas of noticeable deviations between the GT and the PR magnitude maps. (b) Phase of the channel‐wise $B_{1}^{+}$ maps comparing GT data with PR across different sites. Compared to the GTA, A → A performs well across all channels, showing consistent phase estimations. A → B, while largely accurate, shows some deviations to GT, particularly in Channel 1 and Channels 5 and 8, where the phase appears slightly misaligned. Compared to GTB, B → B results are close to the GT, demonstrating strong consistency across all channels. B → A, however, introduces minor but noticeable imperfections in Channels 2–7, which, although minor, are still visible. Compared to GTC, both A → C and B → C diverge from the GT, indicating poor generalization. However, their errors are similar, suggesting that neither model adapts well to GTC, but they remain comparable to each other in terms of deviation. The arrows indicate areas of noticeable deviations between the GT and the predicted $B_{1}^{+}$ maps.

Figure 2 compares $B_{1}^{+}$ magnitude/phase between GT and PR for a representative transversal slice from one subject. As expected, PRs shows the highest qualitative similarity to GTs when prediction and training sites match (A → A outperforms A → B, and B → B outperforms B → A). For $B_{1}^{+}$ magnitude PR, key differences are observed in the smoothness, continuity, and scaling of predictions, particularly in high‐intensity Tx/Rx channel regions. At Site C, magnitude PRs generated with CNN A appear smoother, whereas CNN B tends to produce a more “watery” (murky) continuity, with attenuated magnitudes around the high‐intensity regions. For the phase PR, B → A exhibited reduced smoothness and continuity, as well as phase offsets in certain regions (e.g., Tx2), compared to A → A. In contrast, A → B showed less coherent phase transitions overall (e.g., Tx4) and larger absolute phase errors compared to B → B. Notably, both A → C and B → C phase PRs demonstrated lower accuracy than their respective GTs, highlighting the challenges of generalization to unseen data from new sites.

### 3.2 Error Maps

**FIGURE 3:**
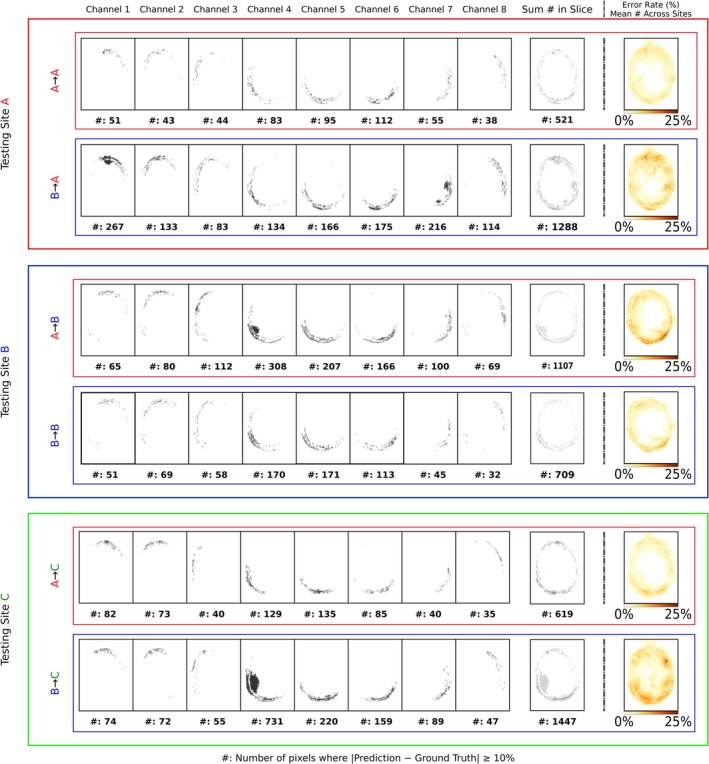
Error maps generated by thresholding the absolute difference between ground truth (GT) and predicted (PR) $B_{1}^{+}$ maps using an arbitrarily chosen threshold of ± 0.1 deviation (i.e., |GT − PR| > 0.1). Voxels exceeding this threshold are highlighted. The color coding is red for Site A, blue for Site B, and green for Site C. Columns 1–8 channel‐wise error maps for one representative slice; Column 9 displays the summed error maps across all channels for that slice. Column 10 presents the mean error map across the full dataset. The error maps of all Tx channels and slices have been accumulated and normalized with the total number of maps for the respective test sites. Total error counts are included below each column.

Figure 3 shows the spatial distribution of $B_{1}^{+}$ magnitude prediction errors exceeding ±10% (in a.u.) of the corresponding ground truth value for each Tx channel, along with the summed error map across all Tx channels for one representative slice. The last column displays the mean error map computed across the entire dataset, that is, across all slices and all Tx channels, normalized by the total number of maps to yield values between 0 and 1.

Figure 3 illustrates that for A → A, mean errors are primarily concentrated along skull edges, with accuracy observed within the center of the brain, and a total error count of 521 for the slice displayed. In contrast, cross‐site B → A shows 2.4‐fold increase in total error count for the same slice as A → A, with Channel 1 reaching a maximum of 267 errors (factor of ~5 compared to A → A). While both A → A and B → A errors are mainly located at the skull edges, B → A additionally exhibits errors within the brain interior. The B → B evaluation shows inferior performance compared to A → A, with a total error count of 709. Despite this higher count, B → B errors, similar to A → A, are primarily located at the skull edges, and the variability in error distribution per channel (32–171 pixels, spread factor of ~5) resembles that of A → A (43–112 pixels, spread factor of ~3). However, A → B shows less absolute errors (1107) compared to B → A (1288), as well as a reduction relative to its baseline B → B with a 1.5‐fold increase. Similarly to B → A and A → A, the A → B errors are mostly concentrated at the skull edges, with only isolated cases (in Channel 4) extending slightly into the brain. A → C errors are almost exclusively located at skull edges, with the center brain voxel values largely falling below the threshold. Lastly, B → C error maps show higher error magnitudes, highlighted by the black spots, than A → C, with 2.3 times more pixels exceeding the ±0.1 threshold. Overall, most discrepancies also occurred at skull edges.

### 3.3 Residual Spread Analysis

**FIGURE 4:**
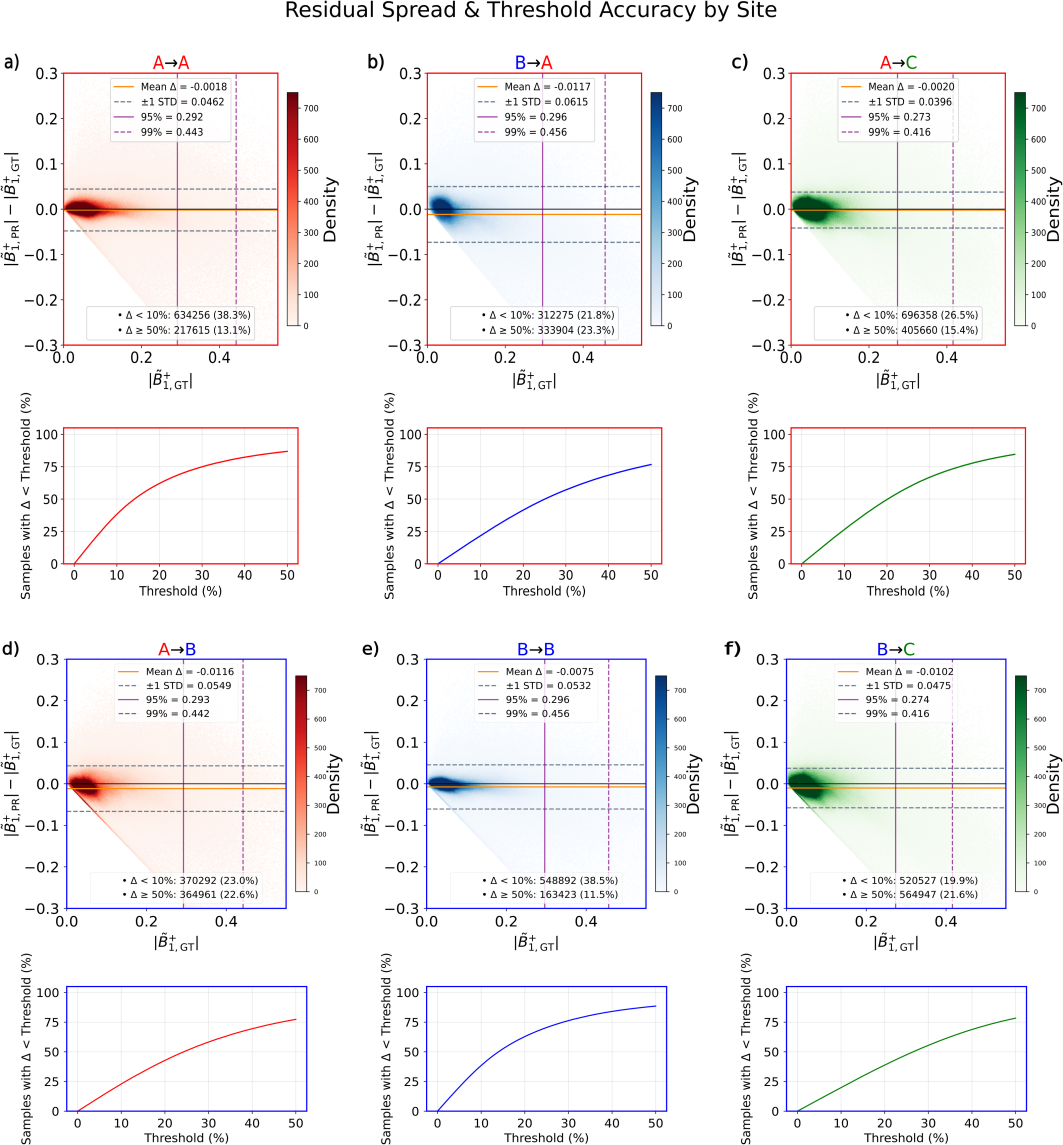
Residual spread plots (top row) and error threshold curves (bottom row) are shown for transversal plane model outputs A → A, B → A, A → C, A → B, B → B, and B → C. In the top row, residuals (*Δ* = |PR| − |GT|) are plotted against ground magnitude values, with point density visualized via color shading. Each plot includes mean ± 1 standard deviation as well as the 95th and 99th percentile residuals, indicated by purple vertical lines to mark extreme deviations and highlight the tail behavior of the error distribution. Below each plot, summary statistics are provided: the number of samples with zero residual, and the number of samples with relative errors below and above 1%. In the respective bottom rows, cumulative threshold curves illustrate the proportion of samples with relative errors below increasing thresholds. These curves offer a comprehensive view of prediction fidelity across the entire range, showing how much of the model output falls within acceptable error bounds.

Figure 4 provides a detailed quantitative analysis of the CNN's performance for outputs A → A, B → A, A → C, A → B, B → B, and B → C using residual spread plots and error threshold curves (for the transversal plane). The y‐axis represents the residual $\Delta =|{\tilde{B}}_{1,\mathrm{PR}}^{+}|-|{\tilde{B}}_{1,\mathrm{GT}}^{+}|$ of the respective $B_{1}^{+}$ maps (normalized, in a.u.) while the x‐axis displays the absolute GT values. Horizontal reference lines were added to mark the mean residual $\bar{\Delta }$ and ±*σ* with *σ* denoting the standard deviation. The top row's Bland–Altman plots show that the highest density of points for all outputs concentrates near *Δ* = 0, indicating alignment between predictions and GT. All outputs maintain a 95th percentile residual distribution below *Δ* = 0.35, with A → A, A → C, B → A, and B → C further achieving a 99th percentile below *Δ* = 0.45. The bottom row's curves reveal distinct patterns: A → A and B → B exhibit a steep initial rise, while B → A shows a more linear initial increase with a potential slope change around the 30% relative error threshold, and B → C displays the most linear form, indicating a more uniform increase in cumulative samples as the relative error threshold rises.

### 3.4 Correlation Evaluation

**FIGURE 5:**
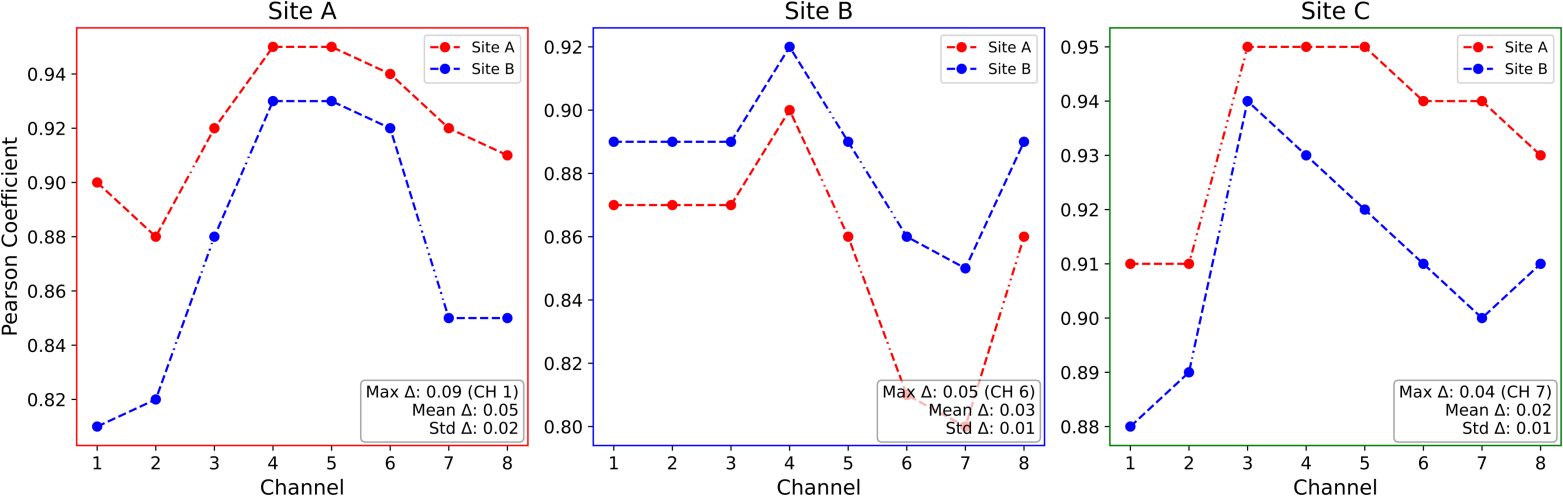
Pearson correlation values per transmit channel of transversal plane. A → A and A → B both show a rising pattern from Channels 1 to 4, peaking at Channel 4 (A → A: 88 → 95, A → B: 81 → 93), then remaining high across Channels 5–6. B → B and B → A follow a similar structure, with Channel 4 again marking the highest score (B → B: 85 → 92, B → A: 80 → 90). These structural similarities align with both pairs using the identical coil model #1.

Figure 5 presents the channel‐wise Pearson's correlation coefficients (for the transversal plane) across all eight transmit channels for each testing site. The patterns between A → A and A → B, as well as B → B and B → A, are similar, although shifted by a mean of 5% and 3%, respectively. This similarity is most evident in the ranking and relative magnitudes of the correlation scores across channels. In both A → A and B → A, Channels 4, and 5 show the highest correlations (A → A: 0.95, 0.95; B → A: 0.93, 0.93), while Channels 1 and 2 have the lowest. Similarly, in B → B and A → B, Channel 4 stands out (B → B: 0.92, B → A: 0.90), while Channels 6 and 7 are lowest (B → B: 0.86, 0.85; A → B: 0.81, 0.80). Comparing the channel‐wise Pearson correlation coefficient differences, a maximum of 0.9 (9%) was found between A → A and B → A for Channel 1 (mean error [9% ± 2%]). A difference of up to 0.5 (5%) was observed between B → B and B → A at Channel 6 (mean [5% ± 1%]). In contrast, the A → C and B → C scores (both using coil #2) did not exhibit a strongly defined pattern, with A → C showing high values broadly distributed across channels and B → C displaying less pronounced channel‐to‐channel variation, resembling more A → B/B → A. Nevertheless, both configurations consistently identify Channel 3 as the best‐performing and Channel 1 as the weakest. The maximum channel wise difference of the Pearson correlation coefficient between A → C and B → C was 0.4 (4%). The overall analysis highlights consistent trends across sites, with Channels 2 and 7 generally having the lowest and Channel 4 the highest correlations.

Additional correlation analyses between predicted (PR) and ground truth (GT) $B_{1}^{+}$ magnitude values across all eight transmit channels are provided in Supplementary Material (Supplementary Fig. App. II.1).

### 3.5 RMSE and SSIM

**FIGURE 6:**
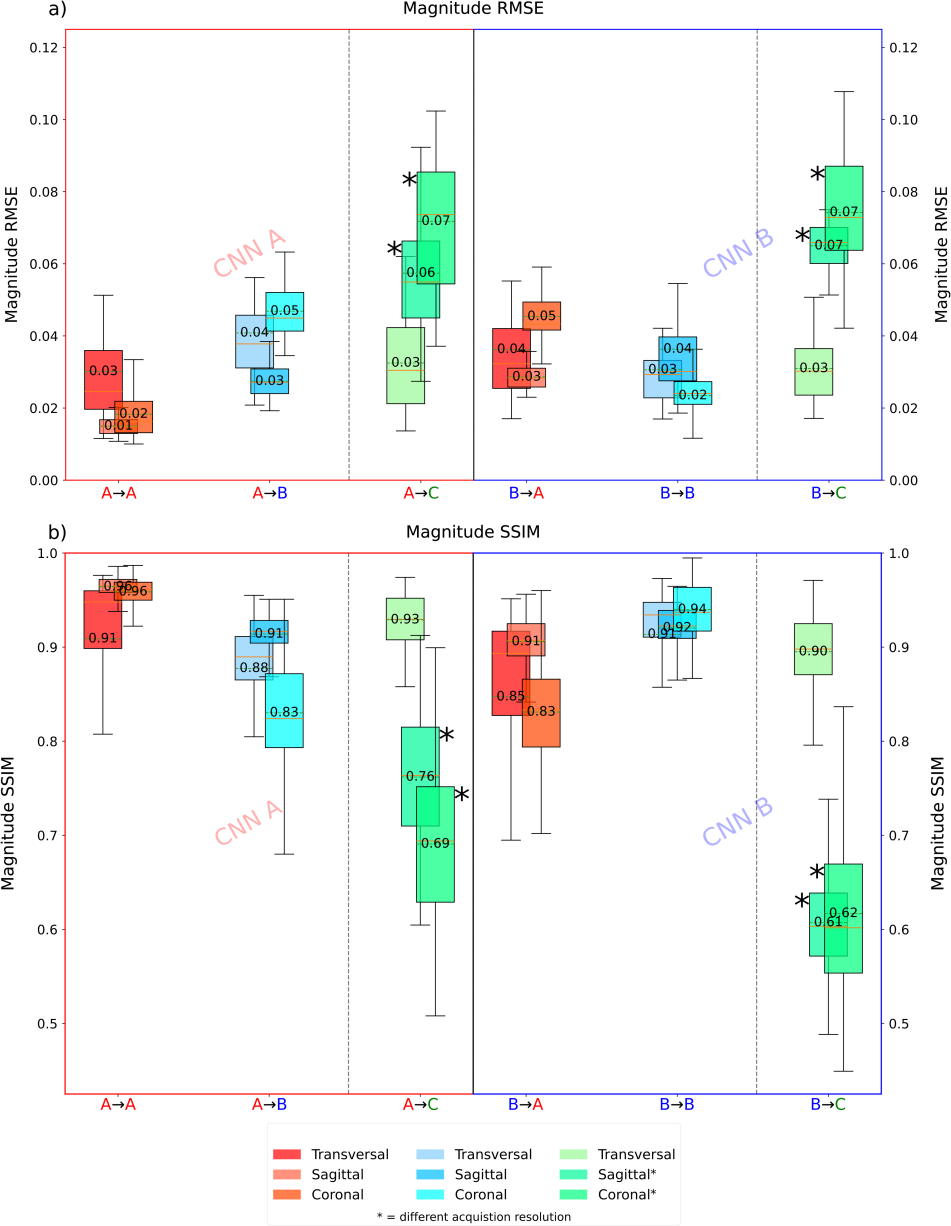
(a) RMSE values for magnitude, predictions across all locations and orientations. Each CNN performs best at its training site (with the solely exception of sagittal A → B outperforming B → B), with consistent and comparable performance trends in absolute values on unseen data. RMSE values for magnitude across all orientations, both for same‐site and cross‐site evaluations of CNN A and CNN B. Full numerical results for RMSE (magnitude, mean phase deviation, imaginary part, complex number) are provided in Supplementary Table S1. (b) SSIM scores for amplitude, predictions across all orientations and training configurations. The layout mirrors that of the RMSE plots for direct comparison. Meanwhile, CNN B outperforms CNN A in the transfer case. Full numerical values for all orientations and additionally for phase and imaginary part SSIM scores are provided in Supplementary Table S1. Note that, for testing C, the sagittal and coronal orientations were acquired at a different resolution and are they are shaded in the table.

Figure 6a presents RMSE scores in boxplot form for magnitude images across all subjects and slices in all orientations. Figure 6b shows the corresponding SSIM scores, mirroring the RMSE boxplot layout to allow direct comparisons between training configurations. Table 2 summarizes the above mentioned results numerically. Additionally, Supplementary Table S1 summarizes all reported metrics, including RMSE for magnitude, phase, imaginary, and complex images (Supplementary Fig. App. II.2 a–d); SSIM for magnitude, phase, and imaginary components (Supplementary Fig. App. II.2 e–f); and PSNR. For each test site, the best‐performing model is highlighted in bold.

**TABLE 2:** Summary of numerical results for magnitude RMSE, SSIM score. Both networks CNN A and CNN B were tested on‐site and cross‐site as well as on Site C. For each testing site, the best‐performing model is highlighted in bold.

Numerical results						
RMSE						
RMSE_MAG_	CNN A			CNN B		
	A → A	A → B	A → C	B → A	B → B	B → C
**Tra**	**3.0% ± 1.6%**	4.1% ± 1.9%	3.2% ± 1.2%	3.6% ± 1.6%	**3.1% ± 1.4%**	**3.1% ± 0.9%**
**Sag**	**1.5% ± 0.3%**	**2.7% ± 0.5%**	**5.7% ± 1.9%**	2.9% ± 0.3%	3.6% ± 1.6%	6.5% ± 0.6%
**Cor**	**1.8% ± 0.7%**	4.7% ± 0.7%	**7.2% ± 1.8%**	4.5% ± 0.6%	**2.3% ± 0.6%**	7.4% ± 1.5%

SSIM						
SSIM_MAG_	CNN A			CNN B		
	**A** → **A**	**A** → **B**	**A** → **C**	**B** → **A**	**B** → **B**	**B** → **C**
**Tra**	**90.9% ± 10.5%**	87.7% ± 7.8%	**92.9% ± 2.6%**	84.7% ± 12.7%	**91.3% ± 8.3%**	89.5% ± 3.6%
**Sag**	**96.4% ± 1.0%**	91.4% ± 2.3%	**76.2% ± 6.8%**	90.6% ± 2.7%	**92.0% ± 2.4%**	60.7% ± 5.0%
**Cor**	**95.9% ± 1.4%**	83.0% ± 5.5%	**69.1% ± 8.2%**	83.1% ± 4.7%	**94.0% ± 2.9%**	61.7% ± 8.5%

In nearly all magnitude RMSE evaluation, the on‐site testing (A → A and B → B) outperformed cross‐site testing across all orientations. When tested on data from Site C, A → C yielded lower RMSE values across two out of three orientations compared to B → C.

For example, the mean magnitude scores over all orientations for A → A were (2.1% ± 1.2%), whereas B → A testing resulted in a higher overall mean RMSE of (3.67% ± 1%). On‐site testing for B → B showed increased compared to A → A scores with (3.0% ± 1.28%), while the transfer case A → B yielded scores of (3.8% ± 1.2%). The largest deviation among all cases was observed in the A → B transfer for the coronal plane, with an increased RMSE of (4.7% ± 0.7%), compared to B → B with (2.3% ± 0.6%). In contrast the smallest deviation was found in the transverse plane between A → A with a score of (3.0% ± 1.5%) and B → A with an increased score of (3.6% ± 1.6%). Notably, CNN B did not always perform best on data from its own testing site, for instance, the sagittal plane, cross‐site testing B → A achieved lower scores of (2.7% ± 0.5%) than B → B with (3.6% ± 1.6%). When evaluated on Site C data, A → C yielded a mean RMSE score of (5.37% ± 1.66%) across all slices, while B → C had a score of (5.67% ± 1.07%).

Overall, among all comparisons in Table 2 (Supplementary Material III.1), A → X yielded better RMSE results in exactly 50% of cases, while B → X performed better in the other 50%.

SSIM results followed the same trend: same‐site testing (A → A and B → B) produced higher scores than cross‐site configurations. Across all SSIM comparisons in Table 2, A → X was superior in 18 out of 27 cases (66.6%), with B → X better in the remaining 33.3%.

Finally, PSNR (Supplementary Table S1) scores again favored same‐site testing, with A → A > B → A and B → B > A → B, as well as A → C > B → C.

### 3.6 SSIM Distribution Plots and Cross‐Site Comparison

**FIGURE 7:**
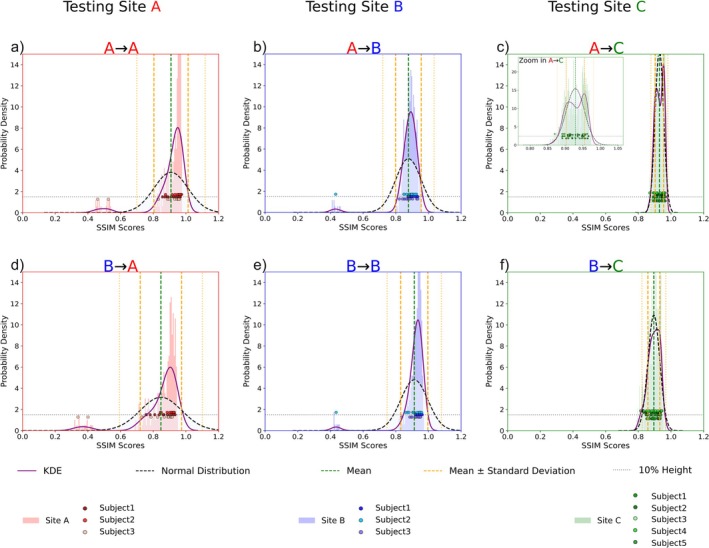
Histogram‐based SSIM distributions for amplitude predictions in the transversal orientation across Sites A, B, and C. Top rows show predictions from CNN A; bottom rows show CNN B. Each histogram includes slice‐wise mean SSIM values (scatter points), a violet KDE curve provides a smooth estimate of the distribution, while a dotted black line represents a Gaussian fit based on the empirical mean and standard deviation. A green dotted line marks the mean, and orange dotted lines denote the first and second standard deviations. The x‐axis ranges from 0 to 1 for SSIM values, and the y‐axis represents normalized probability density. A → A and B → B show compact, high‐scoring distributions with peaks skewed toward higher SSIM values. B → A and A → B display slightly broader with marginally more variable distributions. A → C maintains strong performance, while B → C shows a wider spread and a left‐skewed distribution, indicating lower SSIM values and higher variability across slices.

Figure 7 shows histogram‐based visualizations of SSIM scores for amplitude predictions in the transversal orientation across each evaluation site. Each histogram displays pixel‐wise SSIM scores across the entire dataset, with overlaid scatter points (vertically offset at approximately 10% of the histogram height for visualization) indicating the mean SSIM score for each slice (13 slices per subject), a kernel distribution estimation (KDE) curve fitting the distribution and a Gaussian fit as a comparison. Figure 7a–c show results of A → A, A → B and A → C, whereas Figure 7d–f shows B → B, B → A, and B → C.

For A → A, the distribution shows a sharp peak near the mean, with the KDE curve skewed toward higher SSIM scores, surpassing the Gaussian peak by approximately 50% as indicated by the probability density shown on the y axis. Except for two outliers (in the 0.40–0.60 range) from the same subject, nearly all slice‐wise scores fall within the first standard deviation. The B → A distribution mirrors A → A but exhibits broader KDE and Gaussian curves and a lower peak probability density, indicating higher variability. The B → B distribution mirrors A → A, though with an even sharper peak near the mean. All but one slice lie within the first standard deviation (one outlier at ~0.4). The KDE shows a slight positive skew, with its peak about 50% higher than the Gaussian. In A → B, the distribution is almost an exact mirror of B → B, albeit with a faintly broader KDE. For A → C, the distribution is bimodal with a slight skew toward higher SSIM values. The KDE exhibits a sharper, taller peak than the Gaussian, and most slice‐wise scores are within the first standard deviation, with some extending into the second. The higher subject count for Site C (5 subjects) results in a denser histogram and nearly double the peak probability density of B → C. Finally, the B → C distribution also displays bimodality but with a skew toward lower SSIM values; its left‐hand KDE peak is noticeably higher, and the spread around the mean is wider, with all scatter points evenly distributed.

### 3.7 Tailored pTx Using Rapid DL‐Based $B_{1}^{+}$ maps

**FIGURE 8:**
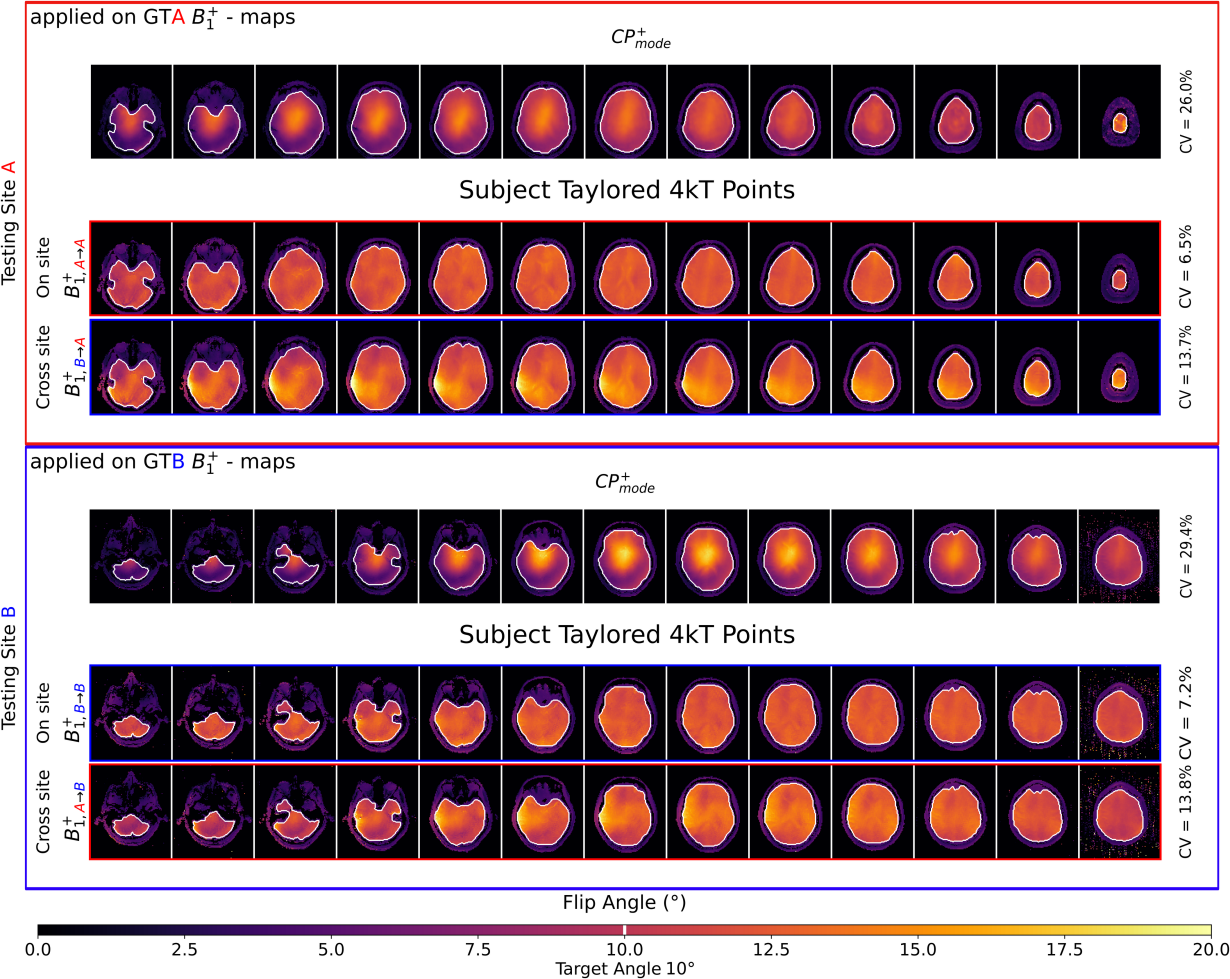
Flip angle (FA) uniformity (with target flip angle of 10°) after Bloch simulation with four different pTx pulses applied to the GT data of two subjects from Testing Sites A and B. The performance of the pulses, tailored on PR $B_{1}^{+}$ maps result in a coefficient of variation (CV) between 6.5% (A → A) and 7.2% (B → B).

Figure 8 summarizes the simulated FA maps using dynamic 4 kT‐points RF pulses for Subject 2 from testing Site A and Subject 2 from Testing Site B, both representative subjects from each site. The depicted FA maps are shown after applying two different 4 kT‐points RF pulses. These RF pulses were calculated [5, 14, 42] using PR $B_{1}^{+}$ maps from CNN A and CNN B, but the final FA maps were evaluated by Bloch simulations using GT $B_{1}^{+}$ maps from Sites A and B, respectively. At Testing Site A, the CV was 6.5% for pulses designed with PR $B_{1}^{+}$ maps from CNN A (A → A), compared to 13.7% for pulses designed with PR $B_{1}^{+}$ maps from CNN B (B → A). At Testing Site B, using GTB $B_{1}^{+}$ maps for the FA evaluation, pulses designed with CNN B predictions (B → B) yielded a CV of 7.2%, whereas CNN A predictions (A → B) produced a slightly lower CV of 13.8%. The results for the other subjects are summarized in Table 3. Shimming on the GT data and applying these solutions to the respective GT data (not shown here) yielded the best performance, with an average CV score of GT A → A: 5.2% and GT B → B: 5.9%. Focusing on the average value across the three subjects, for on‐site PR $B_{1}^{+}$ maps, the average CV scores increased to A → A: 7.2% and B → B: 9.3%, while cross‐site transfer cases showed a further rise (B → A: 15.0% and A → B: 15.4%). The standard CP^+^ mode, both on‐site and cross‐site evaluations resulted in considerably higher CV values (GT A: 24.9%|GT B: 29.4%).

**TABLE 3:** CV reports for Subjects 1–3 (1st column; rows 4–7) at Sites As (Column 2) and B (Column 3), using pulses designed from the PR from CNN A and CNN B when applied on GTA|GTB. Columns 2 and 3 are further divided into two subcolumns: Column 2 reports the CV values for pulses designed with A → A and B → A, then applied to GTA, while Column 3 reports the CV values for pulses designed with B → B and A → B, then applied to GTB. For each testing site, the best‐performing model is highlighted in bold.

Flip angle CV (coefficient of variation) of different tailored 4 kT‐points RF pulses								
Bloch simulation using GT	GT A		GT B		GT A	GT A	GT B	GT B
4 kT points pulses tailored for PR:	A → A	B → A	B → B	A → B	No shim	GT A → A	No shim	GT B → B
	CV	CV	CV	CV	CV	CV	CV	CV
**Subject 1**	**6%**	15.2%	**8%**	19.9%	22.4%	**5.1%**	28.2%	**5.5%**
**Subject 2**	**6.5%**	13.7%	**7.2%**	13.8%	26%	**4.5%**	29.4%	**5.7%**
**Subject 3**	**9.1%**	16.2%	**12.7%**	16.4%	26.3%	**6.2%**	30.6%	**6.6%**

## 4 Discussion

This study examines cross‐site generalizability and transfer performance of CNNs for predicting $B_{1}^{+}$ maps from GRE localizer images in vivo at 7T. Data were acquired at three sites using three different 7T MRI systems and two commercially available 8Tx/32Rx head coils of the same model. While minimizing $B_{1}^{+}$ mapping time is a critical challenge in UHF MRI and has been addressed by CNN‐based approaches in previous work [34, 35], this study focuses on a standardized, multi‐site experimental setup that facilitates data transferability and supports the development of generalizable, site‐independent CNN‐based $B_{1}^{+}$ mapping.

To better assess model performance, we examined the error maps presented in Figure 3. For on‐site cases (A → A, B → B), discrepancies were mainly confined to the skull edges, where the voxels are dominated by bone and fat. However, in these regions, the ground truth $B_{1}^{+}$ maps are typically less accurate and show more outliers as compared to the brain itself. Cross site cases (A → B, B → A) showed additional (1.5‐fold and 2.4‐fold) errors, indicating reduced transferability, most notably in the case of B → A. For A → C and B → C, errors remained mostly at the skull, although B → C showed some intrusion into brain regions. Thus, overall, the network appears to predict well the same‐site $B_{1}^{+}$ maps and tends to yield higher discrepancies in cross‐site predictions.

In addition to the error maps, we used residual spread plots, Pearson's correlations, and PSNR to assess global prediction accuracy. RMSE and SSIM served as our primary metrics. Comparisons for the same‐site (A → A vs. B → B) showed absolute RMSE differences ranging from 0.1% to 2.1%, depending on the orientation, and cross‐site (A → B, B → A) yielded increased but similar differences between 0.2% and 0.5%. Even when applying two different coils, that is, A → C and B → C, the RMSE showed average differences between 0.1% and 1.2%. While these metrics are standard in the field, they appear limited when applied to complex‐valued or spatially structured data on their own. For example, although differences in the error maps among sites are observed (Figure 3), the average RMSE results were largely consistent across sites.

While the RMSE is a more direct measure of predictive accuracy, we also used the SSIM to evaluate prediction quality from a visual and structural perceptual standpoint, thus complementing the RMSE. Since $B_{1}^{+}$ maps contain almost no tissue structure, the SSIM evaluates the structure of the smooth $B_{1}^{+}$ magnitude and phase variations of the different channels and compares them to the $B_{1}^{+}$ fields of the GT in addition to luminance. This employed dual‐metric approach allows for a more comprehensive evaluation of the models as to applying the RMSE alone.

To further investigate and identify sources of error across different sites, we performed a distributional analysis of SSIM values. This involved comparing dataset histograms with KDE curves and fitting Gaussian distributions to approximate prediction accuracy. This approach not only allowed us to assess the average performance but also variability and the presence of outliers, thereby identifying potential systematic errors related to subjects, sites, or acquisition artifacts. For instance, the visualizations of performance differences in Figure 7 revealed bimodal SSIM distributions, suggesting subject‐specific effects. Unfortunately, using the current size of the training and validation datasets, we could not directly correlate these observations with anatomical differences (such as gender or BMI) or known acquisition parameters, indicating that subtle, site‐specific factors such as amplifier settings or scanner operators may also play a role.

Hardware appears to impact the CNN's prediction, even within identical coil models. Input data from Sites A and B (acquired with coil #1) yielded more consistent channel‐wise correlations in intrasite (A → A, B → B) as cross‐site (A → B, B → A) predictions. Conversely, Site C input data (acquired with coil #2 on a 7T Terra Fit) resulted in greater channel variability across CNNs (A → C, B → C) as compared to tests conducted on Sites A and B input data, possibly due to subtle differences in coil and scanner hardware affecting the resulting transmit sensitivities. Notwithstanding these hardware‐induced differences in correlation patterns, Channel 4 consistently demonstrated superior performance, while Channel 2 was consistently the weakest, with Channel 2 positioned on the upper left and Channel 4 on the lower left skull side.

Ultimately, despite the minor differences, the proposed networks effectively predicted channel‐wise $B_{1}^{+}$ data from multiple sites. The suitability of the DL‐based $B_{1}^{+}$ maps was investigated in the context of subject‐specific pTx by optimizing the FA homogeneity in the human brain using dynamic pTx pulses [14, 35]. Such pulses have been calculated based on the estimated whole brain $B_{1}^{+}$ maps (PR) and evaluated using Bloch simulations based on the GT $B_{1}^{+}$ maps. In the best on‐site case, pulses designed with the PR maps achieved a CV of 6%, nearing the performance of pulses designed and evaluated on the GT, which had a CV of 5.1%. While the average CVs across all subjects were higher (A → A: 7.2%; B → B: 9.3%; A → B: 15.4%; B → A: 15.0%), there appears to be a consistent pattern of CV values roughly doubling for unseen sites. This indicates that transferability is feasible but comes at the cost of reduced accuracy compared to on‐site cases. Nevertheless, both on‐site and cross‐site evaluations yielded substantially better results than the standard CP^+^ mode (Site A: 24.9%; Site B: 29.4%). The remaining elevated CV values are likely due to residual deviations between PR and GT. Moreover, issues related to data acquisition at Site B (discussed below) may have contributed to the observed variability. Lastly, a larger training dataset might be needed to better capture the full range of anatomical differences among subjects.

Nevertheless, the outcome of this study opens several promising future opportunities. One potential development is the ability to train a single network that can estimate $B_{1}^{+}$ maps from quickly acquired in vivo localizer images, trained with data from one independent site, and be deployed across multiple sites, without the need for additional data acquisition or retraining. Furthermore, to overcome residual errors in cross‐site predictions the network could be fine‐tuned with a small dataset from the unseen site, improving its initial accuracy. In addition, if a coil is widely used at multiple sites, a large database of $B_{1}^{+}$ maps from different sites can be generated and other sites purchasing the same coil can use the network with little or no new training data. This would enhance the practicality and scalability of the approach, and it increases the dissemination and validation of pTx techniques.

Notwithstanding the previously mentioned results and possibilities, there are also limitations to this study. There is potential for further optimization; for instance, performance could likely be enhanced by fine‐tuning the final network layers for new sites or by conducting an exhaustive hyperparameter search to improve training convergence. However, these steps were considered beyond the scope of this work. Our primary objective was to establish the fundamental feasibility of cross‐site generalization with standard architecture, for which the selected parameters proved sufficiently robust and stable enough. Additionally, integrating a physics‐informed model [32, 43, 44, 45, 46, 47] or more specialized loss functions tailored to specific task optimizations might increase the prediction accuracy.

To further improve the network's performance, a larger training dataset could prove to be beneficial, potentially leading to reduced mean RMSE, higher SSIM scores and less variance between predicted and ground truth $B_{1}^{+}$ maps. In addition, pooled trainings with training data from multiple sites could yield improved on site‐scores as shown in (Tables 1 and 2). However, they reflect an on‐site performance optimum rather than true cross‐site generalization, which was the aim of this study. Furthermore, 2.5D CNN architecture yielded marginal performance improvements compared to the used 2D architecture, but they are affected to interslice spacing, effectively reducing the number of usable slices and complicating cross‐site consistency. A fully 3D architecture could mitigate this limitation and further improve performance but would also require interpolation of the GRE input data and would thus beyond the scope of this study. As suggested in Figure 7 and in Supplementary Material (Supplementary Fig. App. IV.1–IV.4), which shows closely matching curves for coronal and sagittal slices, Gaussian distributions could be used to approximate the SSIM distribution across a dataset. Since a Gaussian curve is well‐defined by its mean and variance, we would predict that increasing the training dataset size could also enhance reliability and predictive stability, suggesting that the model's metrics could be effectively scaled.

While the performance is expected to improve with a larger training library, the approach for the human head requires less training data than previous work on the human body [34, 35]. This effect might be explained by the higher SNR, absence of respiratory and heart motion, and more similar head shapes across volunteers as compared to the body, also considering BMI and gender.

A further point of investigation could be operator‐dependency in the results. In this work, different operators have performed the scans at different sites. To minimize procedural deviations, however, all operators were experienced MR professionals following a standardized protocol; therefore, we are confident that the proposed networks can be applied consistently across various operators and sites. On the other hand, this variability also reflects a realistic scenario in practice, where differences between operators are common and should be accommodated by robust models.

The overall performance differences observed in cross‐site evaluations may be attributable to several issues encountered during data acquisition. First, subject positioning varied between Site A and Site B despite following the same positioning instructions, and even across sessions within Site A. This variability may have contributed to the reduced transfer performance observed during the 4 kT‐point calculation, as illustrated in Figure 8 (positioning).

While head positioning and motion are known to influence $B_{1}^{+}$ field homogeneity and pTx performance [33, 48], our proposed approach demonstrated stable cross‐site transfer in our 4 kT‐point simulations. This suggests a degree of robustness to realistic variations in head size and placement though future work utilizing proprietary coil models for systematic EM simulations, as demonstrated by Plumley et al. [33] could further isolate these effects.

Second, the GRE images from Site B exhibited an overall intensity scaling factor of approximately five compared to those from Site A. Although such scaling is not inherently problematic, because the resulting $B_{1}^{+}$ maps are relative in nature, it nonetheless reflects systematic differences between sites. Finally, an additional experimental artifact was encountered at Site B that required manual correction. In about 40%–45% of the Tx‐channel‐wise GRE acquisitions a random phase offset error of unknown origin led to a mismatch between the complex sum of the individual Tx‐channel‐wise images and the GRE image acquired with all Tx channels transmitting in CP^+^ mode, which served as a reference for consistency check. Such phase errors were not observed at Site A, although the identical GRE sequence was used. To account for this random phase offset in Site B data, we applied a manual voxel‐wise phase SVD‐based correction of the Tx‐channel‐wise images using the GRE image acquired with all Tx combined as a phase reference. A complex‐valued least squares fit was performed to determine the weights that best reconstruct the reference from the individual Tx contributions. To avoid correcting noise‐induced phase fluctuations, only weights with phase angles above ±1° were corrected. The resulting correction factors were then applied to the combined Tx‐channel signals, yielding phase‐corrected complex images. The corrected complex images were subsequently used as CNN input for Site B. In addition, in site B's $B_{1}^{+}$ maps, we observed outliers in the GT data, where the GT values deviated from the expected GT data. These outliers were mostly single pixels and accounted for less than 0.001% of the complete dataset. These outliers were not further accounted for in the analysis.

Lastly, while previous studies [35, 39, 40] have reported improved performance using complex‐valued convolutions and activations, our preliminary tests did not reveal any significant differences (Table 1 and Supplementary Table S2). We therefore limited our study to the more common complex‐as‐channel approach.

Despite these limitations, the present work demonstrates the feasibility of site‐independent $B_{1}^{+}$ prediction using standardized DL methods. Some on‐site PR results are nearly indistinguishable from the GT; for example, the tailored pTx pulses yield CV differences of less than 1% (A → A) and 1.5% (B → B). While the transfer results leave room for improvement in some areas, the approach marks a meaningful step toward more generalizable and automated solutions. These findings help strengthen the methodological basis for developing robust UHF MRI protocols and may support the long‐term goal of broader clinical integration.

## 5 Conclusion

This study demonstrates that CNNs can effectively predict $B_{1}^{+}$ maps across multiple 7T MRI sites using same/identical RF pTx coils. Despite variations in the scanner model and coil hardware, the CNN‐based approach showed promise for robust application across diverse locations. The predicted $B_{1}^{+}$ maps can be effectively used for dynamic pTx highlighting the potential of CNN‐based $B_{1}^{+}$ ‐ estimation techniques as a fast, transferable and scalable solution for UHF MRI workflows. Future work could include implementing additional $B_{1}^{+}$ mapping techniques, as discussed, and expanding the training dataset to improve generalization. A potential next step, although beyond the scope of this work, would be to (cautiousl explore the application of this approach to patient data in a preclinical setting, moving beyond the healthy volunteer cohort used in this study to assess its viability and limitations under more clinically relevant conditions.

6

**Symbols**
X → Y: CNN trained with data from Site X applied to localizer input from Site Y
η: learning rate (used in training setup)
*σ*
: standard deviation
*Δ*
: residual difference (|PR| − |GT|)

## Author Contributions

All authors contributed to the conception of the study, with K.H., F.F.Z., C.S.A., and S.S. leading the conceptualization; data curation and acquisition were performed by K.H., F.K., J.A.G., C.S., L.T.R., K.D., and M.L.; formal analysis and investigation were carried out by K.H. and F.F.Z.; methodology was developed by K.H., F.F.Z., F.K., and C.S.A.; project administration and supervision were provided by S.S., C.S.A., T.S., G.J.M., and M.E.L.; funding was acquired by S.S. and T.S.; the original draft of the manuscript was written by K.H., C.S.A., and S.S., and all authors reviewed, edited, and approved the final manuscript.

## Funding

This study was funded by the Deutsche Forschungsgemeinschaft (DFG, German Research Foundation) (SCHM 2677/4‐1, SCHM 2677/5‐1, and GRK2260, BIOQIC).

## Supporting information


**Data S1:** Supporting Information.

## Data Availability

The data supporting the findings of this study are openly available in Zenodo at https://doi.org/10.5281/zenodo.18338273. The code used for data processing, model training, and evaluation is available at GitHub at https://github.com/hkimon/B1P_Mapping_CCN.git.
