# Supplementary material for: Cross‐Site Generalization of CNN‐Based B1+ Mapping in UHF MRI
Source: NMR Biomed. 2026 Apr 6;39(5):e70263. doi: 10.1002/nbm.70263 (PMC13051333; doi:10.1002/nbm.70263)
Supplement: Supplementary file 1 — Data S1: Supporting Information. [file NBM-39-e70263-s001.docx]

Supplementary Material

# Cross-Site Generalization of CNN-Based $B_{1}^{+}$Mapping in UHF MRI

Kimon Hadjikiriakos^1^, Felix Krüger^1^, Felix Frederik Zimmermann^1^, Johannes A. Grimm^2,3^,
Constantin Schorling ^2,3^, Max Lutz^1^, Simon Schmidt^3,5^, Layla Tabea Riemann^4^,
Katja Degenhardt^1^, Tobias Schäffter^1^, Mark E. Ladd^2^, Gregory J. Metzger^5^,
Christoph Stefan Aigner ^1,6^, Sebastian Schmitter^1,2,5^

*^1^Physikalisch-Technische Bundesanstalt, Berlin, Germany*

*^2^Medical Physics in Radiology, German Cancer Research Center (DKFZ), Heidelberg, Germany*

*^3^Faculty of Physics and Astronomy, Heidelberg University, Heidelberg, Germany,*

*^4^Institute for Applied Medical Informatics, University Medical Center Hamburg-Eppendorf (UKE), Hamburg, Germany*

*^5^Center for Magnetic Resonance Research, University of Minnesota, Minneapolis, Minnesota, USA*

*^6^Max Planck Research Group MR Physics, Max Planck Institute for Human Development, Berlin, Germany*

**Supplementary Material I**

**1.)**

**Input Data: GRE Localizers**

The raw GRE data was first transformed from k-space into image space using a 2D Fast Fourier Transform (FFT). Since the slices were acquired in an interleaved fashion, custom reordering was applied to reconstruct anatomically consistent image stacks.

Noise was quantified using a dedicated noise scan, from which the mean noise level was calculated for each Rx channel. The data were then normalized across channels by dividing the signal magnitude by the corresponding noise estimate. This step improved the signal-to-noise ratio (SNR) and ensured uniform scaling.

For certain tasks like phase alignment, receive channels were also combined using singular value decomposition (SVD), where the first left singular vector represented the dominant coil sensitivity map (CSM). This combination was performed voxel-wise and allowed for the generation of a representative composite image per Tx channel. However, this step was only used for reference computations and not for CNN training.

For CNN input, the full set of 32 Rx channels was preserved after preprocessing and stacked along a further dimension, resulting in complex-valued 3D images per Tx configuration. These multi-channel volumes served as the input to the CNN, which was trained to predict the corresponding transmit channel-wise $B_{1}^{+}$field maps.

**2.)**

**Target Data: Tx-Channel-Wise B1⁺ Maps (Hybrid AFI-GRE)**

The goal was to generate spatially resolved, Tx-channel-wise $B_{1}^{+}$ maps that could serve as training targets for the CNN. To this end, a hybrid approach was adopted following the methodology of Van de Moortele et al.

Three main steps were applied: (1) masking, (2) hybrid map computation, and (3) normalization.

1.A mask was generated from the localizer GRE images to isolate regions of interest. To create this mask, the absolute GRE signal was first summed across all Rx channels $R_{i}$​ ​ and all Tx channels $T_{j}$​. at each spatial location (x, y, z). A global threshold was then applied: any voxel whose summed intensity exceeded a fixed fraction λ of the global maximum was included in the mask.

$$\mathrm{Mask}\left( x,y,z \right)=\sum_{R_{i}T_{j}} \left| S\left( x,y,z,R_{i}T_{j} \right) \right|>\lambda\cdot\max\left( \sum_{R_{i}T_{j}} \left| S\left( x,y,z,R_{i}T_{j} \right) \right| \right)$$

Here, $S\left( x,y,z,R_{i}T_{j} \right)$ denotes the complex GRE signal at voxel location (x, y, z) for receiver channel $R_{i}$​ and transmit channel $T_{j}$​. The parameter λ∈ [0,1] is a user-defined threshold factor that determines the sensitivity of the mask. Finally, $\mathrm{Mask}\left( x,y,z \right)$∈ {0,1} represents the resulting binary mask, where a value of 1 indicates that the voxel is considered part of the relevant anatomical region and included in further processing.

2. To construct the hybrid $B_{1}^{+}$ maps, GRE magnitude images from each Tx-channel-specific acquisition were scaled by the absolute flip angle obtained from an AFI scan. This flip angle map was acquired with all Tx channels active in CP⁺ mode using the Actual Flip Angle Imaging (AFI) method.

The hybrid B1⁺ map for transmit channel $T_{j}$ at voxel (x, y, z) was computed as:

$$B_{1,hybrid}^{+}(x,y,z,T_{j} )=\alpha_{\text{AFI}}\frac{S_{T_{j}}\left( x,y,z \right)}{\sum_{j} \left| S_{T_{j}}\left( x,y,z \right) \right|},\quad T_{j}=1,\ldots,N$$

where $\alpha_{\mathrm{AFI}}$​ denotes the absolute flip angle derived from the AFI scan, and $S\left( x,y,z,R_{i}T_{j} \right)$ is the GRE signal acquired with only transmit channel $T_{j}$​​ active. This normalization ensured a relative field distribution per channel, with the common phase removed, while the AFI provided an absolute scaling reference.

To ensure channel alignment and consistency, Rx coil combination was applied beforehand using sensitivity-based weighting. This combined image was only used during the target generation process and was not part of the training input.

3. Finally, the resulting $B_{1}^{+}$maps were masked using the previously generated region-of-interest mask and then normalized to a nominal flip angle of 90°. This produced the final training targets.

**3.)**

**Pearson correlation coefficient**

The Pearson correlation coefficient r is used to quantify the linear correlation between the predicted and measured values, where r =1 r = 1 r=1 indicates perfect agreement (points lie exactly on the identity line). Despite the visual density of the scatter plots due to the large sample size, clear trends emerge in the channel-wise correlation values.

$$r_{\mathrm{xy}}=\frac{\sum_{i=1}^{n} \left( x_{i}-\bar{x} \right)\left( y_{i}-\bar{y} \right)}{\sqrt{\sum_{i=1}^{n} \left( x_{i}-\bar{x} \right)^{2}\sum_{i=1}^{n} \left( y_{i}-\bar{y} \right)^{2}}}$$

**4.)**

**Root Mean Square Error (RMSE)**

In this study, **root mean square error (RMSE)** was used as a direct, pixel-wise measure of deviation between predicted $B_{1}^{+}$ maps and the corresponding ground truth. By squaring individual errors before averaging, RMSE assigns greater weight to larger deviations, making it especially sensitive to outliers. While this may amplify the influence of isolated errors, it also ensures that substantial mismatches are not masked by small average differences.

A practical advantage of RMSE over related metrics, such as mean squared error (MSE), is that it retains the same physical units as the target variable. This allows for direct interpretability in the context of $B_{1}^{+}$ amplitude, facilitating clearer understanding of prediction error in absolute terms. RMSE is also widely adopted across fields such as signal processing, imaging, and regression, reinforcing its relevance as a standard metric for evaluating model performance.

$$\text{RMSE}=\sqrt{\frac{1}{n}\sum_{i=1}^{n} \left( {GT}_{i}-{PR}_{i} \right)^{2}}$$

**PSNR:**

The Peak Signal-to-Noise Ratio (PSNR) is a commonly used metric for evaluating the quality of image reconstructions by comparing the similarity between a ground truth and a predicted image. It is defined as:

$$PSNR=10\times log^{10}\left( \mathrm{MA}X^{2}/\mathrm{MSE} \right)$$

where MSE is the mean squared error between the two images and MAX is the maximum possible pixel value (typically 1.0 for normalized data or 255 for 8-bit images). PSNR is expressed in decibels (dB), with higher values indicating better fidelity. In general, PSNR values above 40 dB are considered excellent and typically indicate near-indistinguishable differences, while values between 30 dB and 40 dB are considered good. Values below 30 dB often suggest noticeable reconstruction errors, and values below 20 dB are typically associated with poor image quality.

**5.)**

**SSIM Distribution Analysis: Methodology**

This section outlines the procedures used to analyze the distribution of pixel-wise Structural Similarity Index Measure (SSIM) scores across CNN predictions, going beyond mean values to provide deeper insights into model behavior.

**SSIM Score Computation**

For each predicted image $Y$, the SSIM score was computed against the corresponding ground truth image X using the standard formulation:

$$\text{SSIM}\left( x,y \right)=\frac{\left( 2\mu_{x}\mu_{y}+C_{1} \right)\left( 2\sigma_{xy}+C_{2} \right)}{\left( \mu_{x}^{2}+\mu_{y}^{2}+C_{1} \right)\left( \sigma_{x}^{2}+\sigma_{y}^{2}+C_{2} \right)}$$

Where:

- $\mu_{X},\mu_{\hat{X}}\text{ - local means}$
- $\sigma_{X}^{2},\sigma_{\hat{X}}^{2}\text{ - local variances}$
- $\sigma_{X\hat{X}}\text{ - local covariance}$
- $C_{1},C_{2}\text{ - stability constants}$

**Histogram Construction**

To visualize the SSIM distributions:

- Pixel-wise SSIM scores were calculated for all slices and subjects in the dataset.
- These values were aggregated into histograms, normalized such that the total area under each histogram equals 1.
- The x-axis represents SSIM values in the range [0,1], while the y-axis corresponds to probability density.

**Slice-Level Scatter Overlay**

To capture inter-slice variability:

- **Scatter points** were plotted along the base of each histogram (at 10% of vertical axis height).
- Each point represents the **mean SSIM of a single slice** (13 slices per subject), allowing visual assessment of consistency across anatomical levels.

**Kernel Density Estimation (KDE)**

Each histogram was supplemented by a **Kernel Density Estimate (KDE)** curve, offering a smooth, non-parametric approximation of the SSIM distribution. KDE helps to reveal structural features such as skewness and multimodality that may be obscured by histogram binning.

**KDE Formulation:**

$$\hat{f_{h}}\left( x \right)=\frac{1}{nh}\sum_{i=1}^{n} K\left( \frac{x-x_{i}}{h} \right)$$

With:

- n: number of SSIM samples
- $x_{i}$​: individual SSIM values
- $K\left( u \right)$: Gaussian kernel

$$K\left( u \right)=\frac{1}{\sqrt{2\pi}}\exp\left( -\frac{u^{2}}{2} \right)$$

The **bandwidth** h, which controls the smoothness of the estimate, was selected using **Scott’s Rule**:

$$h=\sigma\cdot n^{-1/5}$$

Where σ is the standard deviation of the SSIM values.

**Gaussian Fit and Reference Lines**

To assess normality:

- A **Gaussian probability density function** was fitted to each SSIM distribution using its empirical mean μ and standard deviation σ:

$$\mathcal{N}\left( x;\mu,\sigma\right)=\frac{1}{\sqrt{2\pi\sigma^{2}}}\mathrm{ex}p \left( -\frac{\left( x-\mu\right)^{2}}{2\sigma^{2}} \right)$$

- A **green dotted line** marks the mean μ.
- **Orange dotted lines** indicate μ ± σ and μ ± 2σ, representing one and two standard deviations, respectively.

**Extended Evaluations**

To generalize the findings, SSIM distributions were also analyzed for:

- **Sagittal and coronal orientations**, and
- The **entire evaluation dataset** (12 subjects per training site).

These additional plots are provided in the supplementary material. Due to the increased sample size and anatomical averaging in non-transversal planes, the SSIM distributions in these settings better approximate Gaussian behavior, consistent with the **central limit theorem**.

**Supplementary Material II**

**1.)**

**Correlation Plots**

**
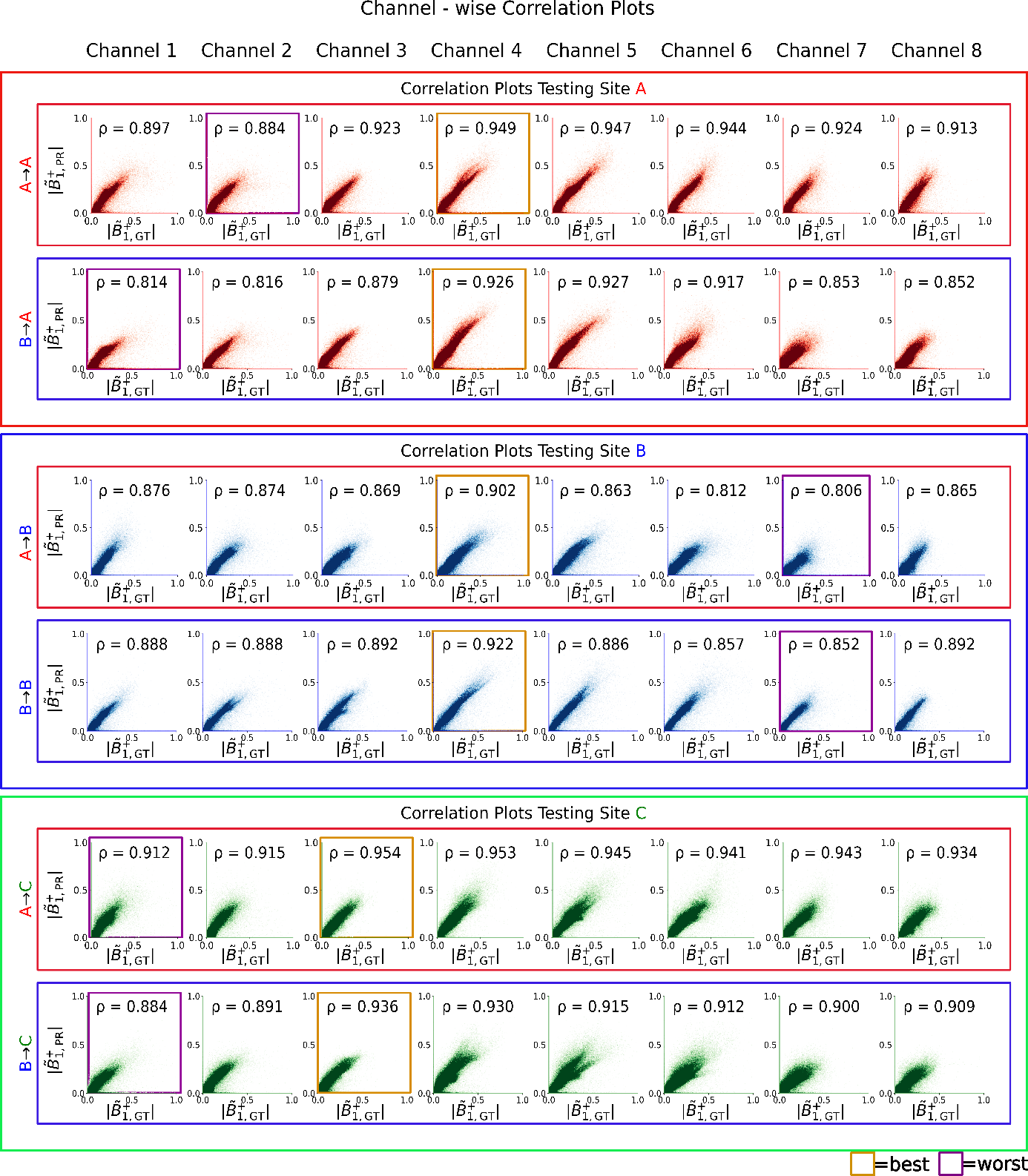
Supplementary Fig. App. II.1**) shows the correlation analysis between PR and GT $B_{1}^{+}$ magnitude values across all eight transmit channels. For A→A, Pearson’s *r* ranged from 0.88 to 0.95, while B→A followed a similar channel wise pattern but with slightly lower values, ranging from 0.77 to 0.90. In contrast, A→B and B→B exhibited weaker performance, with A→B correlations between 0.51 and 0.76, and B→B between 0.73 and 0.88. Across all configurations (A→A, A→B, B→B, B→A), Channel 2 consistently showed the lowest and Channel 4 the highest correlation. While for Site C, B→C preserved this trend with scores ranging from 0.79 to 0.89. A→C exhibited the lowest score with a value of 0.912 at Channel 1, while Channels 3 had the highest score with 0.954. Overall, A→A, B→B and A→C produced higher correlation values compared to B→A, A→B and B→C.

**Supplementary Fig. App. II.1):**  Channel-wise correlation plots, with $|\tilde{B}_{1,PR}^{+}|$on the y axis and $|\tilde{B}_{1,GT}^{+}|$ on the x axis, as well as the Pearson correlation coefficients for models trained on Sites A and B, evaluated on Sites A, B, and C. Models tested on their respective training sites (A→A and B→B) exhibit higher correlation values and tighter clustering, with A→A values ranging from 0.884 to 0.949 and B→B values from 0.691 to 0.875. Cross-site evaluations (A→B and B→A) show lower performance, with B→A value ranging from 0.769 to 0.907 and A→B from 0.52 – 0.757. On Site C, the model trained on Site A (A→C: 0.912–0.954) outperforms the one trained on-Site B (B→C: 0.787–0.892).

**2.)**

**RMSE and SSIM**

**Supplementary Fig. App. II.2 a-d)** present the RMSE scores in boxplot form for: a) magnitude, b) mean phase deviation, c) complex number predictions, and d) the imaginary part, across all subjects and slices in all orientations. **Supplementary Fig. App. II.2 e–g)** present the SSIM scores for: e) magnitude, f) phase, and g) imaginary part, mirroring the RMSE boxplot layout to allow direct comparisons between training configurations. Additionally, Supplementary Table S1 summarizes all discussed metrics, including RMSE, SSIM (for amplitude images), and PSNR, with the best performing model for each testing site highlighted in bold.

For almost all the RMSE testing, the on-site testing (A→A and B→B) performed better in all orientations compared to cross-site testing. The only exceptions were the mean phase deviation scores (7b), where B→A showed less deviation than A→A (B had less deviation). When tested against data from Site C, all orientations of magnitude (7a) and imaginary part (7d) RMSE scores performed better when A→C was applied. For the mean phase deviation (7b) and complex number predictions (7c) RMSE, B→C yielded better results than A→C in all orientations except coronal (7b) and transversal (7c).

For example, the magnitude scores for A→A were 3 ± 1.5%, 1.5 ± 0.2%, and 1.8 ± 0.7% for the different orientations (transversal/sagittal/coronal). When applied to data from Site B, A→B RMSE values increased to 7.8 ± 6.4%, 5.5 ± 4.4%, and 4.8 ± 0.7%. CNN B did not always perform best on data from its own testing site: for example, cross-site testing B→A had improved scores of 4.1 ± 1.6%, 2.9 ± 0.3%, and 4.5 ± 0.6% over B→B with 4.4 ± 1.7%, 3.6 ± 1.6%, and 2.3 ± 0.6%. When evaluated on Site C data, A→C yielded scores of 3.2 ± 1.2%, 5.7 ± 1.9%, and 7.2 ± 1.8%, while B→C had scores of 4.2 ± 5.3%, 2.6 ± 0.9%, and 3.6 ± 1.3%, leading to relative changes in drops of 25%, 14%, and 2.7%. Focusing on the mean phase deviation (7b), it was observed that different orientations had a stronger impact than the choice of training data, except in the case of A→B.

When comparing the SSIM results, the same trend followed as in the RMSE: on-site testing (A→A & B→B) yielded higher scores than cross-site testing, with the sole exception of the SSIM phase (7f) score for the transversal plane, where A→B > B→B. Further, A→C tested better than B→C for all SSIM scores except for the imaginary part in the sagittal orientation (7g). Lastly, when looking at the PSNR scores (Supplementary Table S1), again the on-site testing performed better, with A→A > B→A and B→B > A→B, followed by A→C > B→C.


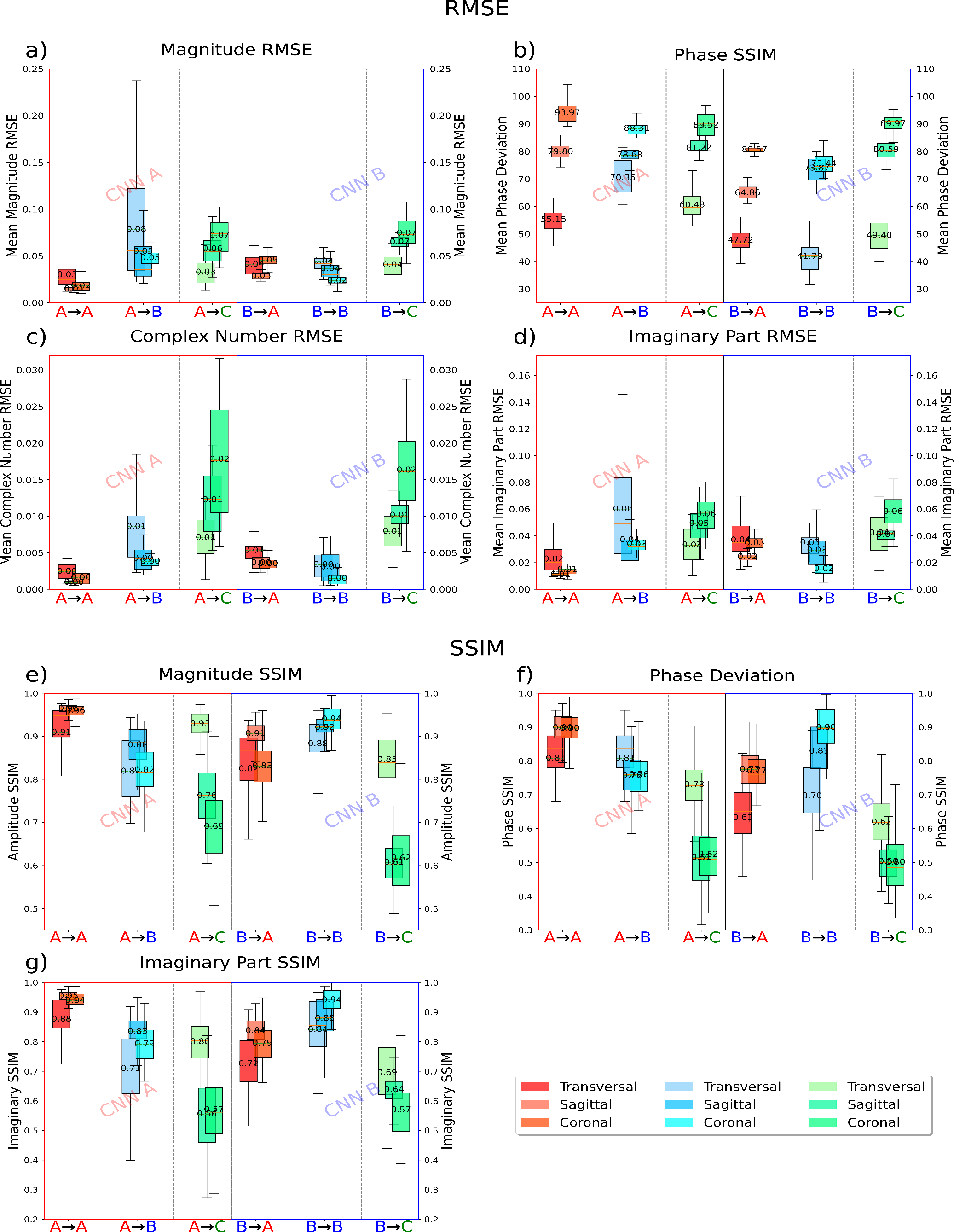


**Supplementary Fig. App. II.2): a-d)** RMSE magnitude, complex, mean phase, and imaginary predictions across all locations and orientations. Each CNN performs best at its training site, with consistent and comparable performance trends in absolute values on unseen data. RMSE values for magnitude, the imaginary part, the complex prediction, and the mean phase deviation follow similar patterns across all orientations, both for same-site and cross-site evaluations of CNN A and CNN B.
Full numerical results are provided in Supplementary Table S1. **e-f)** SSIM scores for amplitude, phase, and imaginary predictions across all orientations and training configurations. The layout mirrors that of the RMSE plots for direct comparison. CNN A consistently outperforms CNN B, especially on-Site C data, with the largest differences in the transversal orientation. While occasional exceptions appear across domains and orientations, the overall trends remain consistent. Full numerical results are provided in Supplementary Table S1

**Supplementary Material III**

**1.) Supportive Tables**

**Supplementary Table S1.**

A full comparison between CCN A and CCN B for RSME (magnitude, mean phase deviation, complex number, imaginary part), SSIM (magnitude, phase, complex number) and PSNR. For each testing site, the best-performing model is highlighted in bold. Supplementary Table S1 provides all numerical results for figure App. II. 2.). In total, A→X yielded better results in 47.3% of cases, and B→X yielded better results in 52.7% of cases for the RMSE scores., A→X yielded better SSIM scores in 18/27 = 66.6% of cases, and B→X yielded better SSIM scores in 33.3% of cases. Looking at the PSNR scores, the on-site testing performed better, with A→A > B→A and B→B > A→B, followed by A→C > B→C.

| **Numerical Results** | | | | | | |
| --- | --- | --- | --- | --- | --- | --- |
| **RMSE** | | | | | | |
| **RMSE_MAG_** | **CNN A** | | | **CNN B** | | |
|  | A→A | A→B | A→C | B→A | B→B | B→C |
| **Tra** | **3.0 ± 1.6 %** | 4.1 ± 1.9 % | 3.2 ± 1.2 % | 3.6 ± 1.6 % | **3.1 ± 1.4 %** | **3.1 ± 0.9 %** |
| **Sag** | **1.5 ± 0.3 %** | **2.7 ± 0.5 %** | **5.7 ± 1.9 %** | 2.9 ± 0.3 % | 3.6 ± 1.6 % | 6.5 ± 0.6 % |
| **Cor** | **1.8 ± 0.7 %** | 4.7 ± 0.7 % | **7.2 ± 1.8 %** | 4.5 ± 0.6 % | **2.3 ± 0.6 %** | 7.4 ± 1.5 % |
| **Mean Phase Deviation** | **CNN A** | | | **CNN B** | | |
|  | A→A | A→B | A→C | B→A | B→B | B→C |
| **Tra** | **55.1 ± 6.0** | 64.38 ± 4.7 | **60.4 ± 4.9** | 66.99 ± 6.0 | **41.8 ± 6.0** | 65.3 ± 5.2 |
| **Sag** | 79.8 ± 2.9 | 76.39 ± 2.7 | 81.22 ± 4.5 | **64.85 ± 2.5** | **73.8 ± 4.6** | **80.6 ± 3.7** |
| **Cor** | 93.9 ± 3.9 | 88.3 ± 2.5 | **89.5 ± 4.2** | **80.6 ± 1.0** | **75.4 ± 3.5** | 89.9 ± 3.3 |
| **RMSE_COMPLEX NUMBER_** | **CNN A** | | | **CNN B** | | |
|  | A→A | A→B | A→C | B→A | B→B | B→C |
| **Tra** | **0.2 ± 0.1 %** | 0.4 ± 0.1 % | 0.7 ± 0.3 % | 0.4 ± 0.1 % | **0.2 ± 0.1 %** | **0.7 ± 0.2 %** |
| **Sag** | **0.1 ± 0.0 %** | **0.3 ± 0.1 %** | 1.2 ± 0.4 % | 0.4 ± 0.1 % | 0.3 ± 0.2 % | **1.0 ± 0.2 %** |
| **Cor** | **0.2 ± 0.1 %** | 0.4 ± 0.1 % | 1.8 ± 0.8 % | 0.4 ± 0.1 % | **0.2 ± 0.1 %** | **1.6 ± 0.6 %** |
| **RMSE_IMAG_** | **CNN A** | | | **CNN B** | | |
|  | A→A | A→B | A→C | B→A | B→B | B→C |
| **Tra** | **2.3 ± 1.1 %** | 2.9 ± 1.2 % | 3.3 ± 1.3 % | 2.8 ± 1.1 % | **2.3 ± 0.9 %** | **3.2 ± 1.1 %** |
| **Sag** | **1.1 ± 0.2 %** | **1.8 ± 0.3 %** | 4.9 ± 1.5 % | 2.4 ± 0.4 % | 2.9 ± 1.4 % | **4.1 ± 0.4 %** |
| **Cor** | **1.5 ± 0.8 %** | 3.3 ± 0.5 % | **5.6 ± 1.2 %** | 3.5 ± 0.5 % | **1.5 ± 0.5 %** | 5.8 ± 1.2 % |
| **SSIM** | | | | | | |
| **SSIM_MAG_** | **CNN A** | | | **CNN B** | | |
|  | A→A | A→B | A→C | B→A | B→B | B→C |
| **Tra** | **90.9 ± 10.5 %** | 87.7 ± 7.8 % | **92.9 ± 2.6 %** | 84.7 ± 12.7 % | **91.3 ± 8.3 %** | 89.5 ± 3.6 % |
| **Sag** | **96.4 ± 1.0 %** | 91.4 ± 2.3 % | **76.2 ± 6.8 %** | 90.6 ± 2.7 % | **92.0 ± 2.4 %** | 60.7 ± 5.0 % |
| **Cor** | **95.9 ± 1.4 %** | 83.0 ± 5.5 % | **69.1 ± 8.2 %** | 83.1 ± 4.7 % | **94.0 ± 2.9 %** | 61.7 ± 8.5 % |
| **SSIM_PHASE_** | **CNN A** | | | **CNN B** | | |
|  | A→A | A→B | A→C | B→A | B→B | B→C |
| **Tra** | **80.8 ± 11.5 %** | 80.8 ± 11.5 % | **72.9 ± 6.6 %** | 69.0 ± 11.4 % | **81.7 ± 9.8 %** | 69.0 ± 6 % |
| **Sag** | **89.8 ± 4.1 %** | 80.5 ± 4.8 % | **51.4 ± 8.9 %** | 77.4 ± 6.1 % | **82.9 ± 8.2 %** | 50.2 ± 5.6 % |
| **Cor** | **89.6 ± 4.3 %** | 76.7 ± 6.3 % | **52.4 ± 8.7 %** | 77.1 ± 5.3 % | **89.8 ± 5.9 %** | 50. ± 9.1 % |
| **SSIM_IMAG_** | **CNN A** | | | **CNN B** | | |
|  | A→A | A→B | A→C | B→A | B→B | B→C |
| **Tra** | **87.6 ± 9.5 %** | 79.8 ± 7.3 % | **79.9 ± 7.4 %** | 79 ± 10.4 % | **88.7 ± 7 %** | 77.3 ± 7.5 % |
| **Sag** | **95.4 ± 1.6 %** | **88.1 ± 3.1 %** | 55.5 ± 11.6 % | 83.7 ± 4.5 % | 87.8 ± 7.4 % | **63.7 ± 4.9 %** |
| **Cor** | **93.9 ± 3.2 %** | 80 ± 6.4 % | **57.1 ± 10.4 %** | 79.5 ± 6.0 % | **94.0 ± 3.6 %** | 56.9 ± 9.4 % |
| **PSNR** | | | | | | |
| **PSNR** | **CNN A** | | | **CNN B** | | |
|  | A→A | A→B | A→C | B→A | B→B | B→C |
| **Tra** | **23.65 dB** | 22.98 dB | **18.51 dB** | 20.68 dB | **25.37 dB** | 17.87 dB |
| **Sag** | **23.16 dB** | 20.80 dB | **13.37 dB** | 16.26 dB | **23.28 dB** | 10.57 dB |
| **Cor** | **18.84 dB** | 11.30 dB | 9.64 dB | 10.88 dB | **19.93 dB** | **10.27 dB** |

**Supplementary Table S1:** Supplementary Table S1 summarizes all evaluated metrics, including RMSE, SSIM (for amplitude, phase, imaginary part, complex number images), and PSNR. For each testing site, the best-performing model is highlighted in bold.

**Supplementary Table S2.**

A full comparison between complex valued CCN A (using L2 Loss) and complex valued CCN B (using L2 Loss) for RSME (magnitude) and SSIM (magnitude) for each testing site, the best-performing model is highlighted in bold.

| **Complex CNN (L2 Loss)** | | | | | | |
| --- | --- | --- | --- | --- | --- | --- |
| **RMSE_AMP_** | **CNN A** | | | **CNN** B | | |
|  | A→A | A→B | A→C | B→A | B→B | A→C |
| **Tra** | **2.8 ± 1.7%** | 4.4 ± 2.1 % | 6.2 ± 3.4 % | **3.6 ± 1.8 %** | 3.3 ± 2.1 % | **6.0 ± 1.6 %** |
| **Sag** | **4.3 ± 1.8 %** | 4.7 ± 2.2% | 6.2 ± 2.1 % | 5.6 ± 2.3 % | **4.5 ± 1.6%** | **5.9 ± 9.2 %** |
| **Cor** | 6.9 ± 4.8 % | 6.7 ± 3.8% | 13.5 ± 12.9% | **5.3 ± 2.0 %** | **5.0 ± 2.3%** | **8.2 ± 4.2 %** |
| **SSIM_AMP_** | **CNN A** | | | **CNN** B | | |
|  | A→A | A→B | A→C | B→A | B→B | A→C |
| **Tra** | **87.7 ± 6.7 %** | 82.2 ± 7.2 % | **64.9 ± 6.5 %** | 82.3 ± 8.9 % | **90.3 ± 5.2 %** | 64.0 ± 6.3 % |
| **Sag** | **92.8 ± 2.6 %** | **87.2 ± 4.7%** | **75.8 ± 8.7 %** | 75.7 ± 6.8 % | 67.7 ± 8.4 % | 44.5 ± 9.2 % |
| **Cor** | **65.8 ± 12.0 %** | **63.1 ± 9.6%** | **55.8 ± 8.7 %** | 51.7 ± 14.2 % | 60.2 ± 9.1 % | 52.2 ± 9.1 % |

**Supplementary Table S2:** Supplementary Table S2 summarizes the RMSE and SSIM scores for amplitude predictions obtained using a complex-valued CNN. For each testing site, the best-performing model is highlighted in bold.

**Supplementary Material IV**

**1.)**

**Supportive Figures – Distributions Plots**

To explore the behavior of the Structural Similarity Index Measure (SSIM) beyond aggregated metrics such as mean and standard deviation, we created histogram-based visualizations of SSIM values in the amplitude domain for the transversal orientation, with separate plots for each evaluation site.

Data Preparation and Slice-Level Analysis

For each evaluation site, predictions from two convolutional neural networks (CNNs) were compared:

- Upper row: predictions from the CNN trained on Site A data.
- Lower row: predictions from the CNN trained on Site B data.

The evaluation was conducted on the same three subjects used for training on Site A and Site B, respectively. This cross-validation scheme ensures balanced subject selection and a controlled comparison between the two networks.

**Supplementary Fig. App. IV. 1**

**Sagittal Subset Distribution**

**
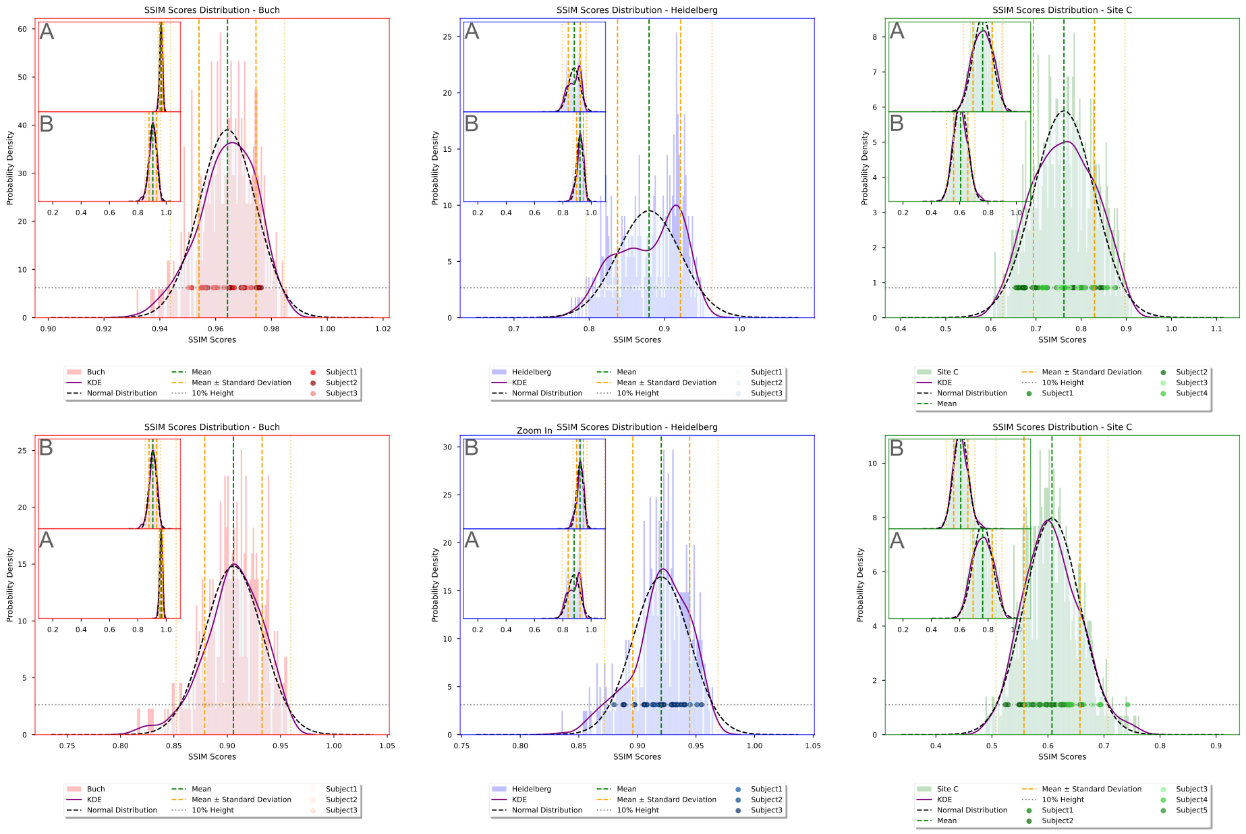
**

**Supplementary Fig. App. IV. 2**

**Sagittal Full Set Distribution**

**
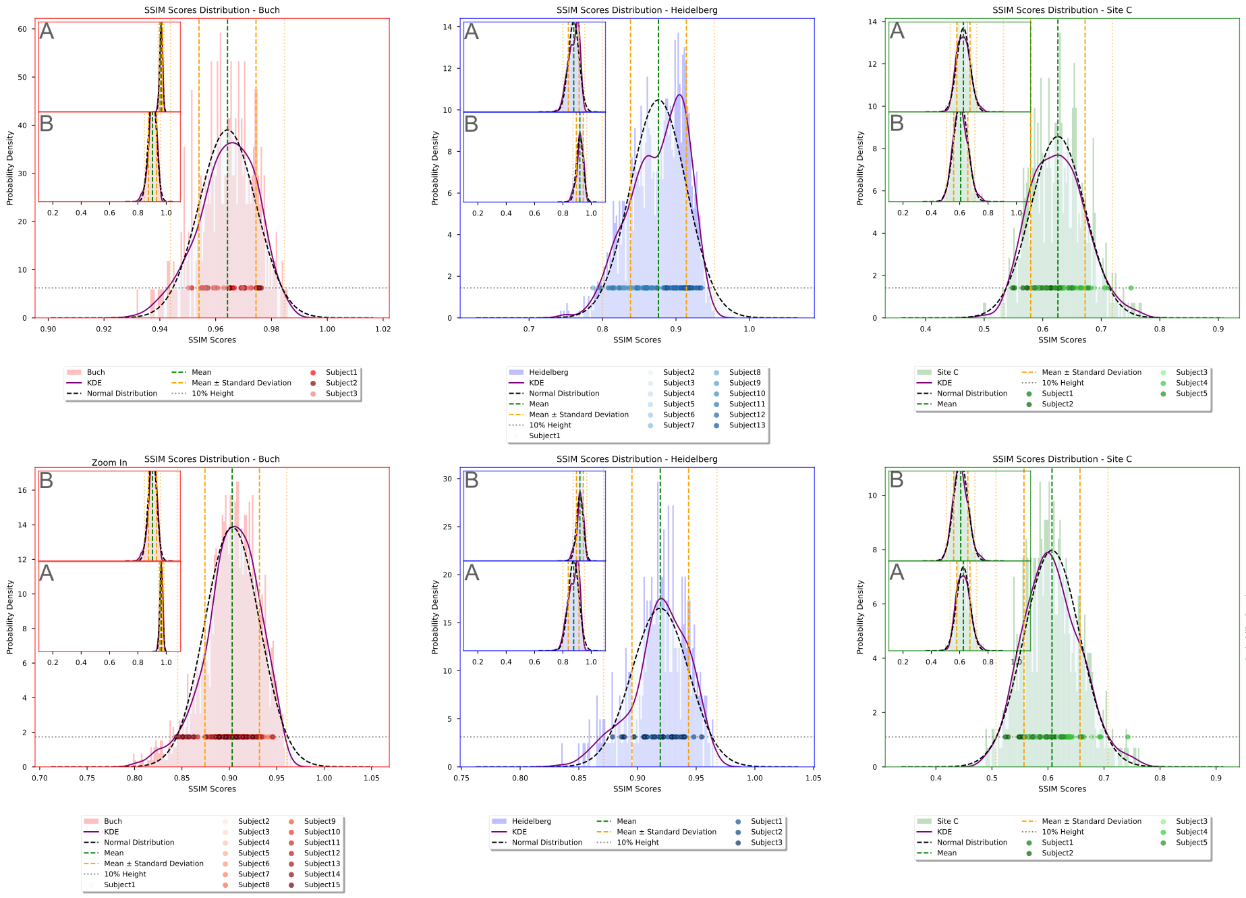
**

**Supplementary Fig. App. IV. 3**

**Coronal Subset Distribution**

**
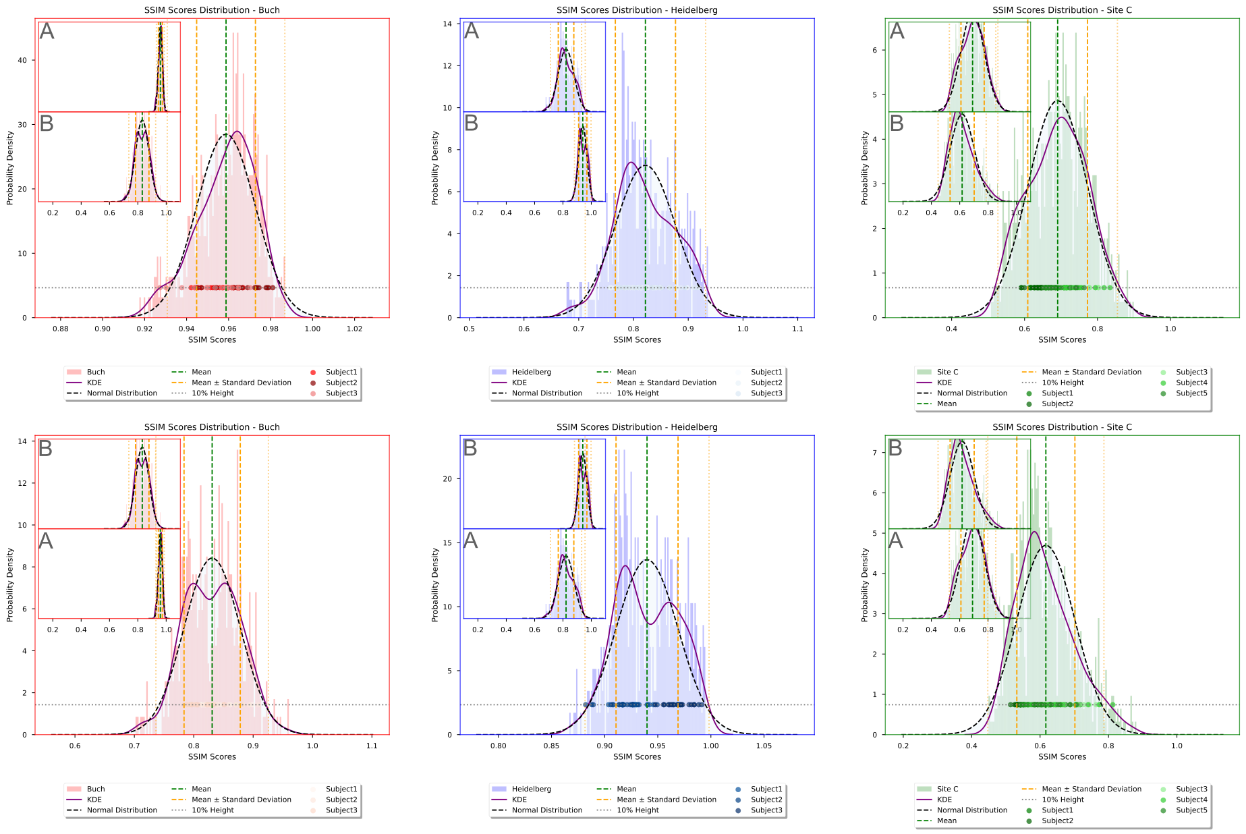
**

**Supplementary Fig. App. IV. 4**

**Coronal Full Set
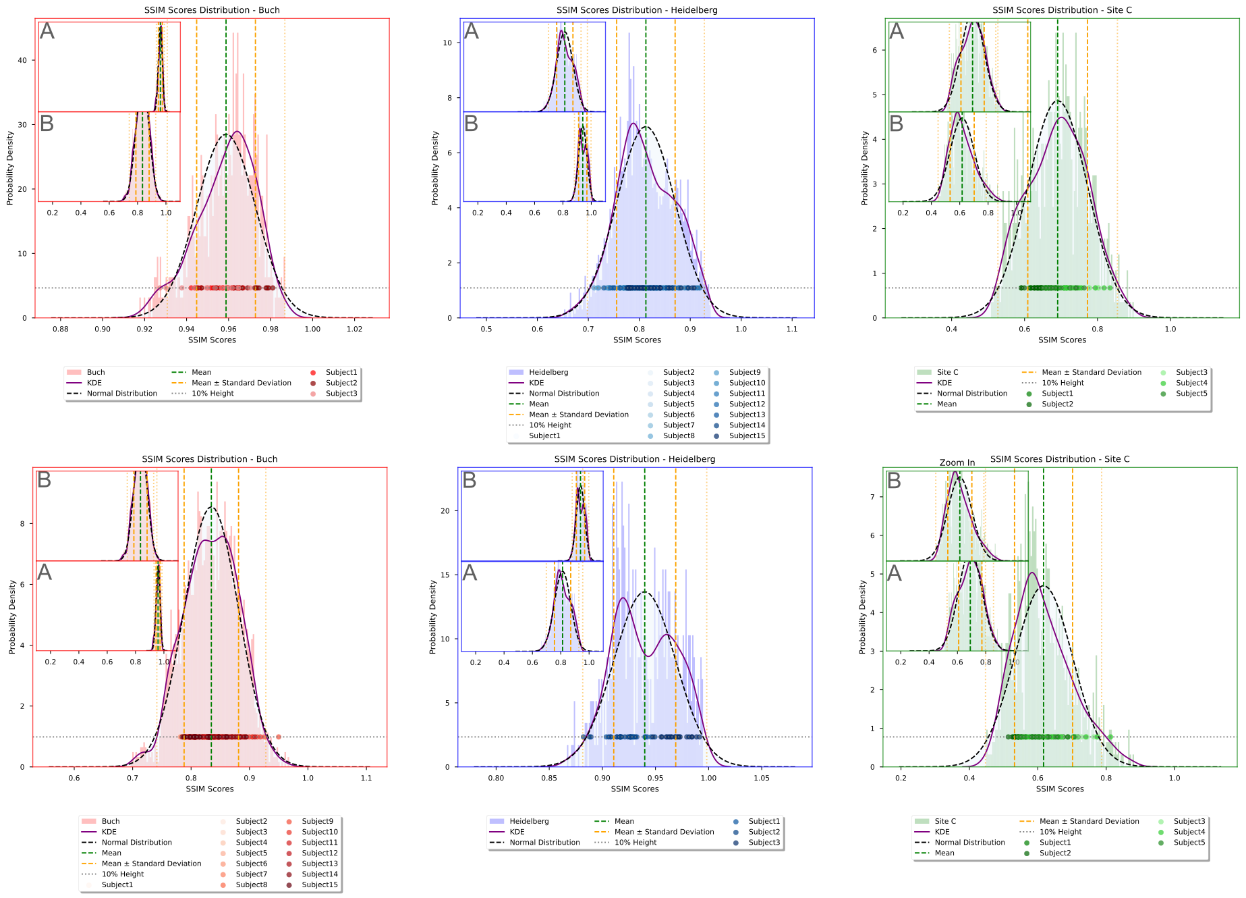
 Distribution**
